# Identification of a conserved gene family with an essential role in *Leishmania* parasite–insect vector adhesion

**DOI:** 10.1073/pnas.2603653123

**Published:** 2026-07-20

**Authors:** Barrack O. Owino, Ryuji Yanase, Katerina Pruzinova, Helen Farr, Yaimie Lopez, Alan O. Marron, Sue Vaughan, Petr Volf, Jack D. Sunter

**Affiliations:** ^a^ Department of Biological and Medical Sciences, Oxford Brookes University, Oxford OX3 0BP, United Kingdom; ^b^ School of Life Sciences, University of Nottingham Medical School, Nottingham NG7 2UH, United Kingdom; ^c^ Department of Parasitology, Charles University, Faculty of Science, Prague 128 43, Czech Republic; ^d^ Sir William Dunn School of Pathology, University of Oxford, Oxford OX1 3RE, United Kingdom

**Keywords:** *Leishmania*, adhesion, parasite–vector interactions, cytoskeleton, flagellum

## Abstract

Adhesion to tissues and surfaces is used by pathogens to avoid clearance, maintain infections, and facilitate transmission. An important example is the adhesion of the *Leishmania* parasite to the stomodeal valve of its insect vector, the sand fly, which is important for life cycle progression, and occurs through its flagellum via a cytoskeletal adhesion complex. While three essential kinetoplastid-insect adhesion proteins (KIAPs) are known, the overall proteome of the adhesion complex is obscure. Using TurboID-tagged KIAP3 combined with proteomics and light microscopy, we have identified additional proteins associated with the adhered flagellum, including multiple members of the Adhesion Related NTPase-like Domain (ARND) family, of which the canonical member is KIAP4, described here. Phylogenetic analysis of the ARND family showed it is conserved across the kinetoplastids, and we demonstrate that *Trypanosoma congolense* paralogs localize to the adhered flagellum in that parasite. Deletion of KIAP4 severely impaired haptomonad adhesion, with the mutant parasites unable to colonize the sand fly stomodeal valve. Our work provides details of the molecular machinery essential for vector colonization and shows that these proteins have a conserved function in other kinetoplastid parasites.

## Significance

*Leishmania*, the causative agent of leishmaniasis, has multiple developmental forms as it alternates between a mammalian host and an insect vector. A key form is the haptomonad that adheres to the stomodeal valve of its insect vector and is important for efficient transmission. Although three kinetoplastid-insect adhesion proteins (KIAPs) are known, the broader molecular machinery mediating adhesion remains obscure. Here, we identify KIAP4 as an essential adhesion-plaque component and a canonical member of a conserved ARND protein family that is found across kinetoplastids. KIAP4 deletion severely impaired haptomonad adhesion and prevented colonization of the sand fly stomodeal valve. These findings reveal key molecular components required for vector colonization, highlighting potential targets for transmission-blocking interventions.

Adhesion to tissues and surfaces is often used by pathogens to avoid clearance and progress their life cycle. *Plasmodium falciparum* sequestration in the vasculature (1) and adhesion of kinetoplastid parasites (e.g., *Trypanosoma brucei*) to insect tissues are classic examples (2–4). For some pathogens, such as *Yersinia pestis* and *Leishmania*, adhesion is complemented by secretion of an extracellular matrix that partially obstructs the gut of their insect vectors, leading to frequent but incomplete feeding and hence enhanced transmission (5–7).

*Leishmania* spp. are unicellular flagellated parasites that cause leishmaniasis, which remains a significant global health problem with a broad geographic distribution and over 1 billion people at risk of infection (8–10). *Leishmania* spp. have a complex life cycle involving a vertebrate host and a sand fly vector, with diverse morphological forms depending on specific ecological niches (11, 12). Infection of the sand fly occurs through ingestion of a blood meal containing the amastigote form. In the sand fly, the amastigotes differentiate into the motile procyclic promastigote form, which undergo multiple differentiation stages, culminating in the generation of mammalian infective metacyclics and the haptomonad form that adheres stably to the sand fly stomodeal valve (6, 7, 12–14). Haptomonads promote *Leishmania* transmission in three ways. First, they contribute to the production of the promastigote secretory gel, which blocks the gut, and along with the damage they inflict on the stomodeal valve, results in increased feeding attempts (7, 12, 13, 15–18). Second, haptomonads can divide asymmetrically to produce motile promastigote forms, contributing to persistent sand fly infections (19). Third, detached haptomonads form an important part of the infectious inoculum (14).

The promastigote is characterized by an elongated cell body and a long, motile flagellum that extends from the anterior cell tip (12, 18, 20). In contrast, the mature haptomonad is characterized by a stumpy cell body and a short flagellum that is enlarged at the tip (4, 18, 19, 21–23). This highly modified flagellum contains a complex cytoskeletal structure comprising an adhesion plaque, which is positioned adjacent to the cuticle surface of the stomodeal valve, and a dense filamentous network that extends from the plaque and connects to the anterior cell tip cytoskeleton (18, 19, 24).

**Fig. 1.**
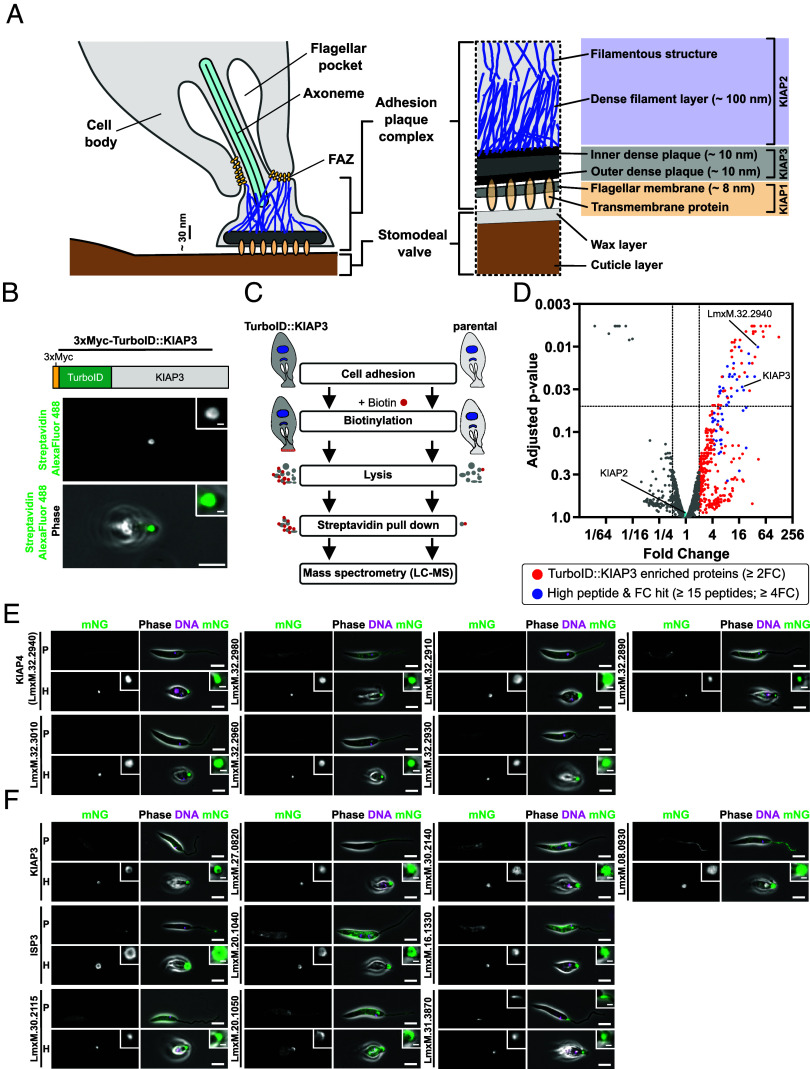
TurboID::KIAP3 proteomics identifies KIAP4 and six other members of the Adhesion Related NTPase-like Domain (ARND) family, Related to *SI Appendix*, Figs. S1–S3, and Datasets S1–S3. (*A*) A schematic illustrating the key structures of the adhered flagellum of *Leishmania* haptomonad cells. The scale bar denotes the approximate thickness of the adhesion plaque (~30 nm). FAZ: flagellum attachment zone. (*B*) Fluorescence micrographs of adhered in vitro haptomonad cells showing the localization of biotinylated proteins to the adhered flagellum. Biotinylation was examined by fluorescence microscopy after the addition of exogenous biotin. Insets show a magnified view of the adhered flagellum. (Scale bar, 5 μm, *Insets*: 1 μm.) (*C*) Workflow of protein sample preparation from TurboID::KIAP3 and parental cell lines. Adhered cells were incubated with biotin for 18 h and lysed, followed by protein enrichment using streptavidin-coated magnetic beads and mass spectrometry. (*D*) KIAP3 proximal proteins identified from two independent technical replicates. Red points indicate proteins enriched by ≥2-fold-change in the TurboID::KIAP3 compared with the parental cell line, while the highly enriched (≥4-fold change) and abundant proteins (≥15 peptides) selected for tagging are shown in blue. The gray points show proteins with limited to no enrichment. The dashed vertical lines show ≤0.5 or ≥2-fold change thresholds, whereas the horizontal line indicates the significance threshold (*P* < 0.05). Pair-wise statistical testing between sample groups was performed using limma via FragPipe-Analyst (28, 29), followed by the Benjamini–Hochberg approach for multiple test corrections. The positions of KIAP3 (bait), KIAP2, and the canonical member (LmxM.32.2940) of the novel Adhesion Related NTPase-like Domain (ARND) family are shown. (*E*) Fluorescence micrographs showing the signals of KIAP4 and six other Adhesion Related NTPase-like Domain (ARND) family members in the promastigote (P) and in vitro haptomonad cells (H) adhered flagellum. Images are representative of two biological replicates. For promastigotes, 50 cells were imaged for each cell line. For mature in vitro haptomonads, a strong adhered flagellum signal was observed in 100% (n = 29 cells) for LmxM.32.2940 (KIAP4), in 100% (n = 28 cells) for LmxM.32.3010, in ~96% (n = 23 cells) for LmxM.32.2980, 100% (n = 39 cells) for LmxM.32.2960, 100% (n = 17 cells) for LmxM.32.2910, 100% (n = 15 cells) for LmxM.32.2930, and 96% (n = 25 cells) for LmxM.32.2890. (*F*) Localization of the other 11 proteins, including three proteins previously associated with haptomonads (KIAP3, ISP3, LmxM.30.2115). Images are representative of 3 biological replicates for KIAP3 and LmxM.31.3870 and 1 biological replicate for the other cell lines. For promastigotes, 50 cells were imaged for each cell line. For mature in vitro haptomonads, a strong adhered flagellum signal was observed in 100% (n = 53 cells) for LmxM.20.1190 (KIAP3), 100% (n = 13 cells) for LmxM.15.0520, 100% (n = 12 cells) for LmxM.30.2115, ~83% (n = 6 cells) for LmxM.27.0820, 75% (n = 8 cells) for LmxM.20.1040, ~76% (n = 13 cells) for LmxM.20.1050, 100% (n = 11 cells) for LmxM.30.2140, 100% (n = 135 cells) for LmxM.31.3870, and ~86% (n = 7 cells) for LmxM.08.0930. The nucleus and kinetoplast DNA were stained with Hoechst 33342 (magenta). The maximum and minimum fluorescence intensity values were adjusted to show the optimal fluorescence signal of each protein in the in vitro haptomonad cell (H), and the intensity values were applied to the respective promastigotes (P). Insets illustrate the details of protein localization in the promastigote and adhered flagellum of in vitro haptomonad cells. (Scale bar, 5 μm, *Insets*: 1 μm.) See also *SI Appendix*, Figs. S1–S3 and Datasets S1–S3.

We previously identified three kinetoplastid-insect adhesion proteins (KIAP1-3) that localize to distinct domains of the adhered flagellum (24) (Fig. 1*A*). KIAP1 is a transmembrane protein, whereas KIAP2 and KIAP3 each contain a catalytically inactive, calpain-like cysteine peptidase domain, likely important for protein–protein interactions (24–27). Both KIAP1 and KIAP3 localize to the adhesion plaque, while KIAP2 is positioned in the filamentous network that extends from the adhesion plaque to the anterior cell tip (18, 24). KIAP1-3 are predominantly expressed in haptomonads and are essential for adhesion and colonization of the sand fly stomodeal valve (24). However, given the complexity of the adhesion plaque and filaments, there are likely additional important components to discover.

Here, we developed a TurboID proximity labeling approach (30, 31) to define additional molecular components of the adhesion plaque. We identified a cohort of components that are associated with the adhered haptomonad flagellum, including multiple members of a gene family that we have termed the Adhesion Related NTPase-like Domain (ARND) family, of which the canonical member is KIAP4. High-resolution microscopy indicated that KIAP4 is a component of the adhesion plaque. Paralogs of the ARND family are found in the related parasite, *Trypanosoma congolense*, and they also localized to the adhered flagellum in this species. Deletion of the canonical member of the ARND family, KIAP4, severely impaired haptomonad adhesion, with the null mutants unable to colonize the sand fly stomodeal valve. Our work provides more details of the molecular machinery essential for vector colonization, opening avenues for developing transmission-blocking strategies across kinetoplastids.

## Results

### TurboID::KIAP3 Proteomics Identified KIAP4 and Six Other Members of the Adhesion Related NTPase-Like Domain (ARND) Family.

To identify additional components of the adhesion plaque in *L. mexicana*, we employed a TurboID approach using KIAP3 (24) as the bait (Fig. 1*A*). We generated cells in which KIAP3 was endogenously tagged with TurboID fused to a 3xMyc tag at the N terminus, referred to as TurboID::KIAP3 (Fig. 1*B* and *SI Appendix*, Fig. S1*A*). To determine whether TurboID::KIAP3 was functional, cell lines expressing TurboID::KIAP3 were allowed to adhere for 72 h and biotinylation was initiated by the addition of exogenous biotin. To assess biotinylation, we probed the cells with streptavidin-AlexaFluor 488, followed by fluorescence microscopy. There was a strong fluorescence signal in the enlarged region of the adhered flagellum, with minimal signal in the cell body (Fig. 1*B*), confirming that the TurboID fusion retains biotin ligase activity and can biotinylate proximal proteins in the adhered flagellum of in vitro haptomonad cells.

To identify the biotinylated proteins, we prepared protein samples from adhered parental cells expressing Cas9 nuclease and T7 RNA polymerase (C9T7) (32) and TurboID::KIAP3 cells, as illustrated in Fig. 1*C*. Adhered cells were lysed and biotinylated proteins enriched using streptavidin-coated magnetic beads, as shown by western blotting for the TurboID::KIAP3-tagged cell line compared with the parental cell line (*SI Appendix*, Fig. S1 *B* and *C*). Mass spectrometry of enriched proteins from both parental and TurboID::KIAP3 cells detected 2,969 proteins across all three technical replicates, of which 450 were enriched by ≥2-fold in TurboID::KIAP3 compared with parental cells (Fig. 1*D* and Dataset S1). These included KIAP3 and a protein family arrayed on chromosome 32 (e.g., LmxM.32.2940 and LmxM.32.2980), which appeared among the top hits. We also detected KIAP2, an essential protein for *Leishmania* adhesion (24), but it was not enriched (Fig. 1*D*). We then applied stringent selection criteria to the 450 enriched proteins in the TurboID::KIAP3 sample, focusing on those proteins with at least 4-fold change enrichment compared with the parental cells and that were likely abundant (≥15 peptides). This resulted in a cohort of 58 proteins, which were carried forward for an endogenous tagging screen (Dataset S2).

We successfully endogenously tagged 55 of these proteins at the C-terminus with mNeonGreen (mNG), except KIAP3, which was tagged on the N terminus, as previously shown (24). The localization of the tagged proteins was examined in promastigote and in vitro haptomonad cells by fluorescence microscopy (Fig. 1 *E* and *F* and *SI Appendix*, Fig. S2 *A* and *B*). The free-swimming *Leishmania* promastigotes have an elongated cell body, with a long, motile flagellum extending from the anterior cell tip (18). Contrastingly, in vitro haptomonad cells have a stumpy cell body, with a short and enlarged flagellum that is adhered to the glass surface (4, 19, 21). Initially, we categorized these proteins depending on whether there was any fluorescence signal present in the adhered flagellum. This approach identified 32 proteins that were present in the adhered flagellum of mature in vitro haptomonad cells (Fig. 1 *E* and *F* and *SI Appendix*, Fig. S2). We then segregated these proteins into those with a strong or weak signal in the adhered mature in vitro haptomonad flagellum (Fig. 1 *E* and *F* and *SI Appendix*, Fig. S2*A*); 17 had a weak signal in the promastigote but showed a strong signal in the adhered flagellum (Fig. 1 *E* and *F*).

Intriguingly, seven of these 17 proteins, including the top hit in our screen, LmxM.32.2940, are all encoded, except one, by an array of genes on chromosome 32, that contain one or more p-loop NTPase domains (Fig. 1*E*). We named the gene family – Adhesion Related NTPase-like Domain (ARND) family and the canonical member (LmxM.32.2940), KIAP4 (Kinetoplastid Insect Adhesion Protein 4), based on its localization and function (see below). The remaining 10 proteins included the known haptomonad proteins, KIAP3 (the bait; LmxM.20.1190), the inhibitor of serine peptidases protein 3 (ISP3; LmxM.15.0520), and LmxM.30.2115 (14, 24, 33) (Fig. 1*F*).

### Phylogenetic Analysis Reveals Ancient Paralogy and Lineage-Specific Expansion of the Adhesion Related NTPase-Like Domain (ARND) Gene Family.

Using KIAP4 and iterative BLAST analyses against the *Leishmania mexicana* proteome, we identified 10 additional Adhesion Related NTPase-like Domain (ARND) gene family proteins, which included the six we had detected by proteomics and localized to the adhered in vitro haptomonad flagellum (*SI Appendix*, Fig. S3*A*). Protein architecture varied across members, but all have a predicted p-loop NTPase domain, with all the proteins having predicted coiled-coil regions and regions of intrinsic disorder (*SI Appendix*, Fig. S3*A*). To determine whether the p-loop NTPase domains were potentially active, we used Foldseek to identify the best match in a common model organism to the AlphaFold3 predicted 3D structure for each p-loop NTPase domain. We then determined if the Walker A-motif was conserved (Dataset S3). Invariably, the best match for ARND gene family proteins was from a kinesin; however, for all the predicted p-loop NTPase domains, the Walker A-motif was not conserved, suggesting these NTPase domains are not active. Hence, we termed these NTPase-like domains.

**Fig. 2.**
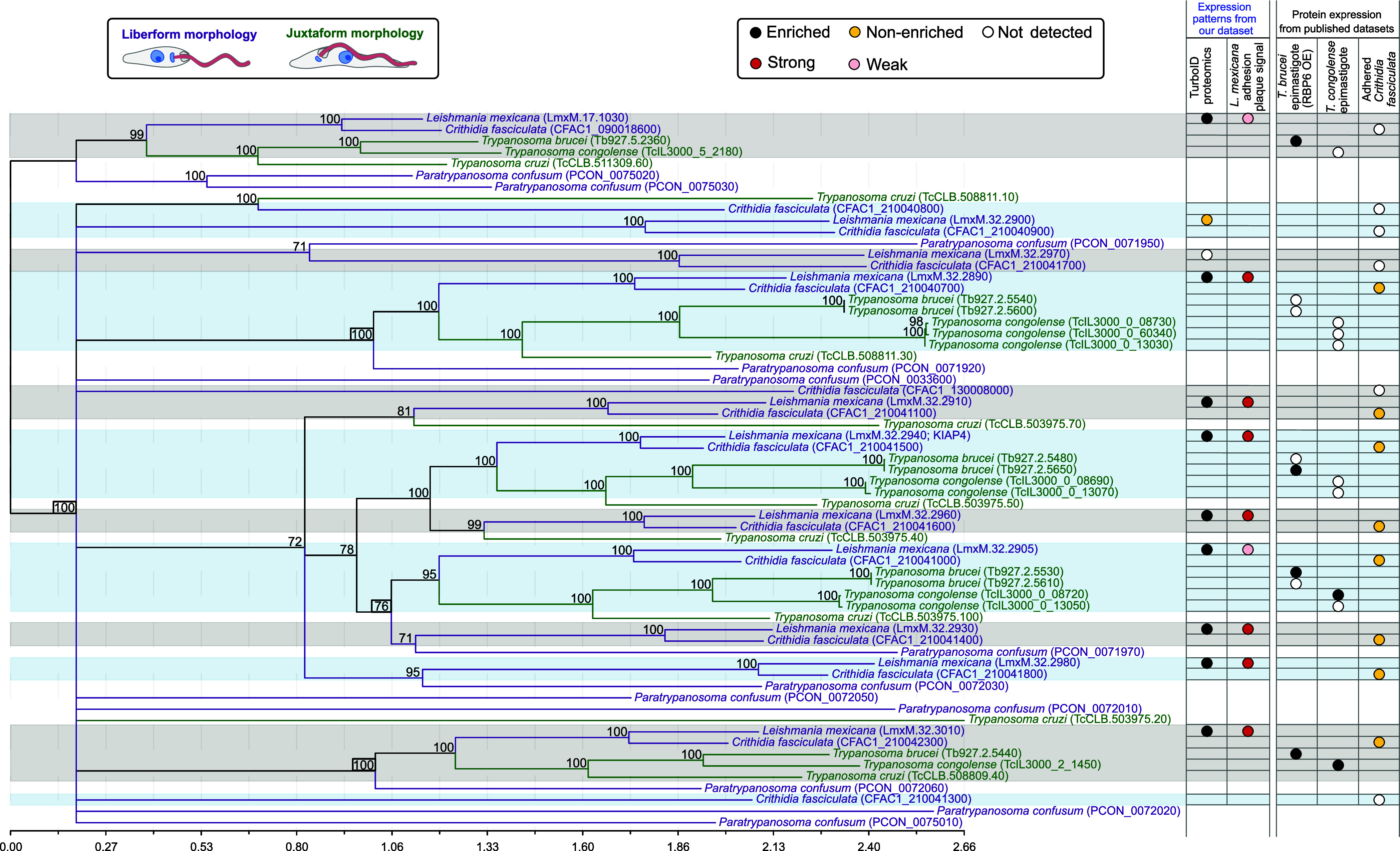
Phylogenetic analysis reveals ancient gene duplication and lineage-specific expansion of the ARND gene family, Related to *SI Appendix*, Fig. S3 and Datasets S2 and S3. Unrooted maximum likelihood phylogenetic tree showing the evolutionary history of Adhesion Related NTPase-like Domain (ARND) gene family across different kinetoplastid species, representing the liberform (shown in purple) and juxtaform (in green) morphological superclasses. The tree was inferred using the best-fitting model (LG+F + G4) implemented in IQtree and PhyML. The branch lengths represent the number of substitutions per site, while the percentage bootstrap values from 1000 replicates are shown next to the branches. There were 63 amino acid sequences and 6472 positions; statistically unsupported branches with bootstrap values <70% were collapsed. Support values for clades containing all six species in the analysis are shown in boxes. The *Right* panel shows the expression patterns and localization of ARND gene family proteins from our dataset and published proteomics datasets associated with RBP6 overexpressing *T. brucei* (35), adhered *T. congolense* epimastigotes (36), and adhered *C. fasciculata* (37). The red and light red filled circles show paralogs with strong or weak localization signals, respectively, in the *L. mexicana* adhered flagellum. The black, orange, and white filled circles represent paralogs that were enriched, nonenriched, or not detected in the datasets, respectively. Enriched proteins were defined as those with ≥4-fold change in TurboID::KIAP3 vs parental cells, or those with ≥2-fold change in adhered forms vs nonadhered forms in published datasets. The blue or gray horizontal shades do not represent clustering patterns. RBP6 overexpression (OE). See also *SI Appendix*, Fig. S3 and Datasets S2 and S3.

To identify such ARND gene family proteins in other kinetoplastids, we performed an iterative BLAST search against the proteome of representative kinetoplastid species (*SI Appendix*, Fig. S3*B*) using the 11 *L. mexicana* proteins as the query. This analysis revealed that while the protein number varied across different kinetoplastid species, all but *Bodo saltans* and *Trypanosoma rangeli* had multiple ARND family genes (*SI Appendix*, Fig. S3*B*). To understand the evolution of the ARND gene family, we constructed a maximum likelihood phylogenetic tree of a subset of kinetoplastid species representing the two morphological superclasses – liberform and juxtaform (34) (Fig. 2). The phylogenetic analysis revealed ancient gene duplication events within the family, indicated by deep branching patterns and high bootstrap support (>75%) for clades containing orthologous genes from all species included in the analysis (Fig. 2). Generally, we observed clades in which proteins from species in both the liberform and juxtaform morphological superclasses were present. However, in addition, several deep branches only contained proteins from liberform species. In three clades, there were multiple *Trypanosoma congolense* and *Trypanosoma brucei* paralogs, suggesting a shared duplication event in their common ancestor.

Six of the nine ARND gene family proteins enriched in our proteomics, in addition to KIAP4, had a strong localization in the adhered flagellum of in vitro haptomonad cells (Fig. 1*E*), suggesting that this family of proteins is likely important for adhesion. We examined published proteomic datasets associated with adhered forms to determine the expression pattern and localization of the ARND gene family paralogs in *T. brucei, T. congolense,* and *C. fasciculata* (Fig. 2). While none of the paralogs were upregulated in adhered *C. fasciculata* in vitro (37), many of the paralogs in *T. brucei* were significantly upregulated at the protein level following RBP6 overexpression that recapitulates the epimastigote form, which in the tsetse fly adheres to the salivary glands (Fig. 2) (35). We also noted that the *T. congolense* paralogs, TcIL3000_0_08720 and TcIL3000_2_1450, were upregulated in epimastigote forms (Fig. 2). The upregulation of the *T. congolense* and *T. brucei* paralogs in the epimastigote forms strongly suggested a role in insect vector interactions.

### ARND Gene Family Paralogs Localized to the Adhered Flagellum in *T. Congolense* Epimastigote Forms.

**Fig. 3.**
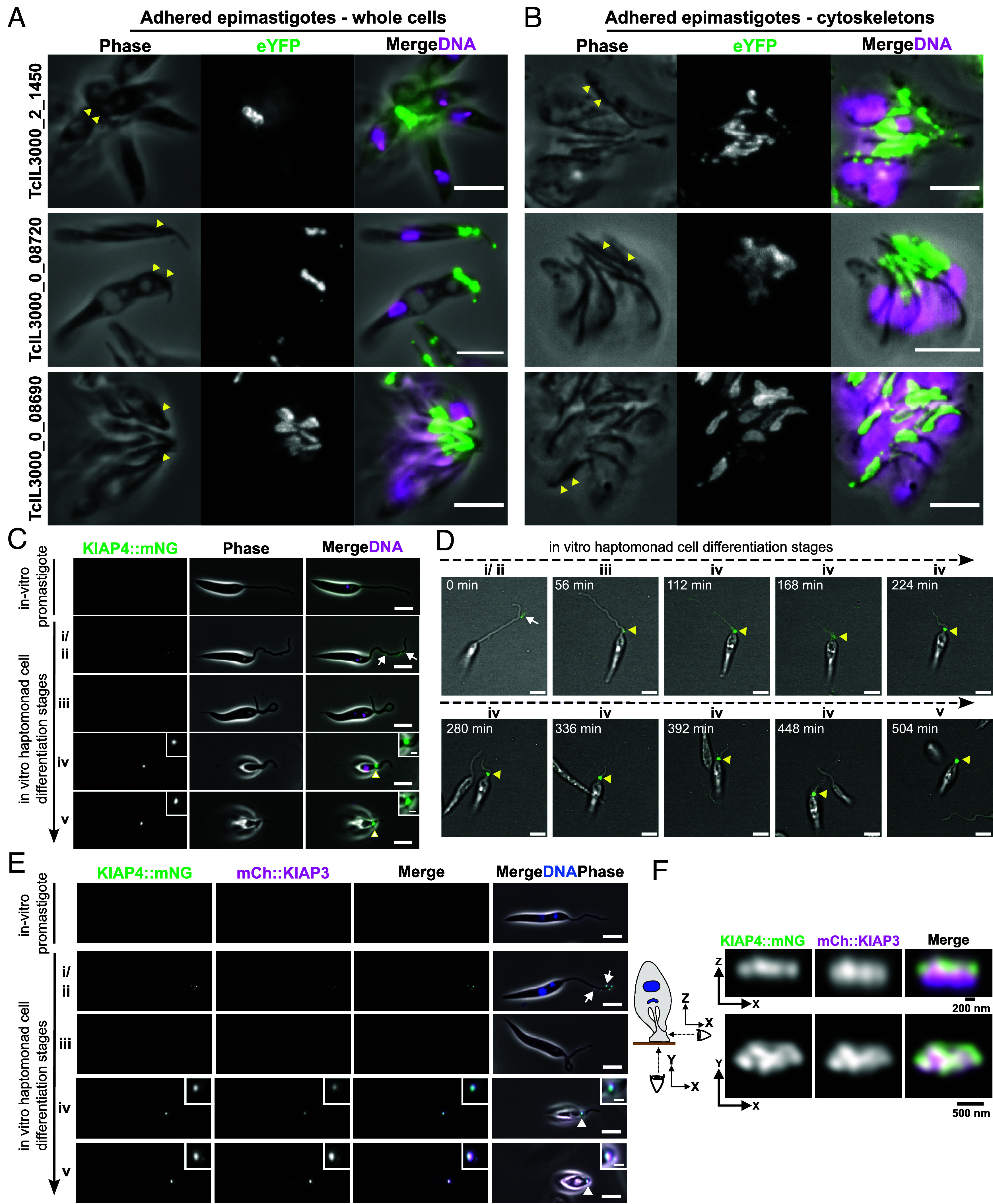
ARND gene family paralogs localize to the adhered flagellum in *Trypanosoma congolense* epimastigote forms, with KIAP4 and KIAP3 having similar development patterns and localization in the *Leishmania* adhesion plaque, Related to *SI Appendix*, Fig. S3 and Datasets S1–S3 and Movie S1. Fluorescence micrographs showing the localization of three eYFP-tagged ARND gene family paralogs in *T. congolense* epimastigote form (EMF). Images of whole cells (*A*) or detergent-extracted cytoskeletons (*B*) from cell lines expressing the three proteins are shown, with the arrowheads (yellow) indicating regions of lateral adhesion via the flagellum. The nucleus and kinetoplast DNA were stained with DAPI, shown in magenta. (Scale bar, 10 μm.) Representative images from n = 50 cells per cell line and for each condition. (*C*) Fluorescence micrographs showing KIAP4::mNG signal at different stages of in vitro haptomonad cell differentiation. The minimum and maximum fluorescence intensities were adjusted to show the optimal KIAP4::mNG signal in the mature in vitro haptomonad cell (stage v), and the intensity values were applied to cells in stages i–iv of differentiation. The nucleus and kinetoplast DNA (shown in magenta) were stained with Hoechst 33342. The arrows show signals at the initial adhesion points, consistent with membrane deformations, while the arrowheads denote the enlarged region of the adhered flagellum. Representative images from two sample preparations are shown. (*D*) Sequential frames at 56 min intervals from a time-lapse movie showing KIAP4::mNG signal development during adhesion. The arrow (white) shows the initial adhesion point, while the arrow heads (yellow) indicate points of stable adhesion and KIAP4::mNG signal accumulation in this region. Representative images from two independent sample preparations are shown. (*E*) Fluorescence micrographs of *L. mexicana* in vitro promastigote and in vitro haptomonad cells expressing mNG-tagged KIAP4 (green) and mCh-tagged KIAP3 (magenta) at different stages of in vitro haptomonad cell differentiation (i–v). The nucleus and kinetoplast DNA were stained with Hoechst 33342 (shown in blue). The maximum and minimum fluorescence intensities were adjusted to show the optimal mCh::KIAP3 and KIAP4::mNG fluorescence signals in the mature in vitro haptomonad cell (stage v), and the intensity values were applied to cells in stages i–iv of differentiation. The arrows show the initial foci of adhesion, while the arrowheads indicate the enlarged region of the adhered flagellum containing an adhesion plaque. Insets show details of protein localizations in the adhered flagellum. Representative images from three independent sample preparations are shown. (Scale bar, 5 μm, *Insets*: 1 μm.) (*F*) Average intensity projected confocal micrographs from 10 Airyscan-processed z-stack slices (1 μm total thickness) of mature in vitro haptomonad cells’ adhered flagellum expressing KIAP4::mNG and mCh::KIAP3. The schematic diagram shows the different viewing positions: side view (X–Z) and bottom view (X–Y). Representative images from five (n = 5) independent sample observations are shown. See also *SI Appendix*, Fig. S3 and Datasets S1–S3 and Movie S1.

We took advantage of the ability to generate adhered *T. congolense* epimastigotes in vitro to determine whether the ARND gene family in this species is involved in parasite adhesion. We generated cells expressing eYFP-tagged versions of three (TcIL3000_2_1450, TcIL3000_0_08720, and TcIL3000_0_08690) of the nine *T. congolense* paralogs and examined their localization in adhered epimastigote forms (Fig. 3 *A* and *B*). *T. congolense* adheres to surfaces through the lateral side of the flagellum (2) rather than the flagellar tip, as observed in *Leishmania* (19). Fluorescence microscopy of adhered whole cell and detergent-extracted epimastigotes revealed concentrated signals along the lateral side of the flagellum in contact with the glass surface for all three proteins (Fig. 3 *A* and *B*). This indicates that these proteins are associated with the adhesion plaque in *T. congolense* and that the ARND gene family members are important for the adhesion of other kinetoplastids to their insect vectors.

### KIAP4 and KIAP3 have Similar Development Patterns and Localization in the *Leishmania* Adhesion Plaque.

As KIAP4 (LmxM.32.2940) was the most enriched member of the ARND family identified by our proteomics, we wanted to investigate its functions in more detail. Our tools to examine adhesion are more advanced in *Leishmania*, so we chose to do it in this organism. We have previously shown by time-lapse microscopy that promastigote to in vitro haptomonad differentiation proceeds through five defined stages, from surface probing and initial localized adhesion via the flagellum in stages i and ii to flagellum disassembly in stage iv and adhesion maturation in stage v (19). To understand the changes in KIAP4 localization during differentiation, we allowed cell lines expressing KIAP4::mNG to adhere to glass coverslips and examined changes in protein signal at different stages of differentiation by microscopy coupled with time-lapse analysis (Fig. 3 *C* and *D*). In promastigotes, KIAP4::mNG localized with a weak signal in the cytoplasm and flagellum (Fig. 3*C*). During differentiation, we observed an accumulation of KIAP4::mNG signal, beginning with bright spots along the flagellum (arrows) in the initial stages (i and ii), consistent with membrane deformations, to an increasingly concentrated fluorescence signal in the enlarged region of the flagellum (arrowheads) in stages (iv and v) (Fig. 3 *C* and *D* and Movie S1). This resembled our previous observations with KIAP1-3 (24).

Given that we detected KIAP4 using KIAP3 as the bait, we next wanted to confirm they were near each other in the adhered flagellum. To address this, we generated cells in which both KIAP4 and KIAP3 were tagged with mNG and mCherry (mCh), respectively, and examined their positions during adhesion (Fig. 3*E*). We observed a similarity in KIAP4::mNG and mCh::KIAP3 localizations, beginning with bright spots along the flagellum in the initial stages of adhesion (Fig. 3*E*). As the differentiation progressed to the final stages, the fluorescence signal of both proteins became concentrated in the enlarged region of the adhered flagellum adjacent to the anterior cell tip (Fig. 3*E*).

To investigate the position of KIAP4 and KIAP3 within the adhered flagellum in more detail, we employed super-resolution confocal microscopy to examine adhered cells in the final stage of differentiation, expressing both KIAP4::mNG and mCh::KIAP3 (Fig. 3*F*). KIAP4::mNG and mCh::KIAP3 had a similar localization, with uneven lateral distribution when viewed through the coverslip (Fig. 3 *F*, *Bottom* panel), and both formed a variegated band adjacent to the glass substratum when viewed from the side, with the KIAP4::mNG signal being slightly narrower than that of mCh::KIAP3 (Fig. 3 *F*, *Top* panel). These findings show that KIAP4 is an adhesion plaque protein and is positioned alongside KIAP3 in the in vitro haptomonad adhered flagellum.

### KIAP4 is Required for Adhesion and Colonization of the Sand Fly Stomodeal Valve.

**Fig. 4.**
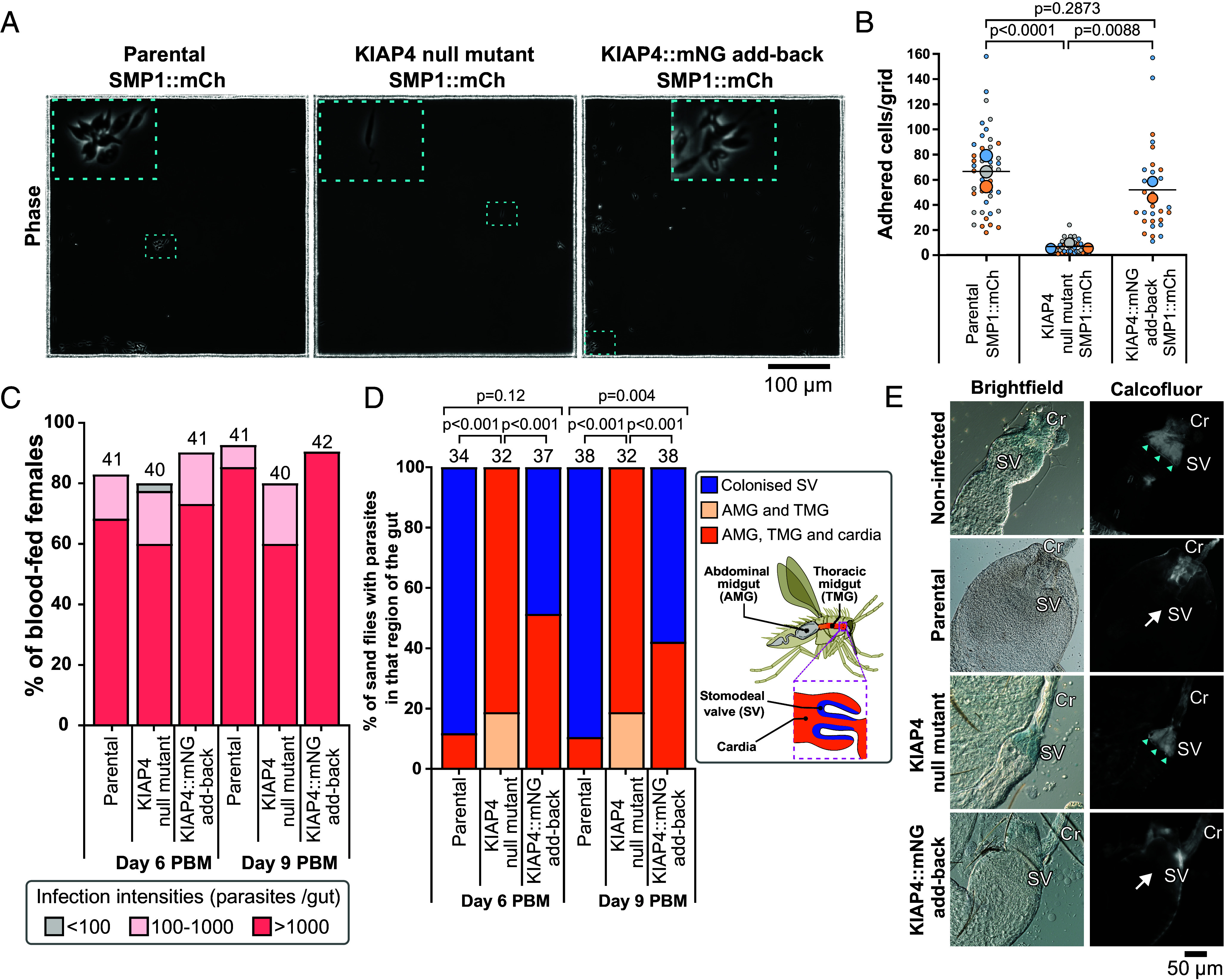
KIAP4 is required for adhesion of in vitro haptomonad cells and colonization of the sand fly stomodeal valve, Related to *SI Appendix*, Fig. S4 and Movies S2–S4. (*A*) Phase-contrast micrographs of *L. mexicana* parental, KIAP4 null mutant, and KIAP4::mNG add-back after 24 h of in vitro haptomonad cell differentiation on gridded glass coverslips. Representative images of adhered cells per grid area for at least 10 grid areas are shown. Insets show an enlarged view of the dotted areas, illustrating examples of adhered cells for the three cell lines. (*B*) Quantification of the number of adhered cells per grid for each cell line. The gray, blue, and orange colors represent measurements from three (for parental and KIAP4 null mutant) or two (for KIAP4::mNG add-back) independent experiments. The larger circle indicates the mean from each independent adhesion experiment and the bar indicates the mean of these means. The *P*-values were calculated using a two-tailed Welch’s *t* test at *P* < 0.05. (*C*) Infection rates and intensities of infections for the parental, KIAP4 null mutant, and KIAP4::mNG add-back cell lines on 6- and 9-d post blood meal (PBM). Values above the bars indicate the total number of examined midguts from two independent replicates. Infection intensities were classified as light (<100 parasites/gut), moderate (100 to 1,000 parasites/gut), and heavy (>1000 parasites/gut). (*D*) Localization of infections for the parental, KIAP4 null mutant, and KIAP4::mNG add-back cell lines on days 6 and 9 PBM. Numbers above the bars indicate the total number of evaluated (positive) female sand flies from two independent replicates. The schematic shows the names and location of the different regions of the sand fly gut. The *P*-values were calculated using a Fisher’s exact test. (*E*) Brightfield and fluorescence images of dissected and calcofluor-stained midguts of noninfected sand flies or sand flies infected with the parental, KIAP4 null mutant, or KIAP4::mNG add-back cell lines from (*A*–*D*) on day 7 PBM. Arrowheads (cyan) show the intact chitin layer in the noninfected and KIAP4 null mutant infected sand flies, while the calcofluor signal was depleted in the sand flies infected with the parental and KIAP4::mNG add-back cell lines (arrows). SV: stomodeal valve; Cr: crop. Representative images of 30 midguts from two independent replicates for each cell line. See also *SI Appendix*, Fig. S4 and Movies S2–S4.

Next, we asked whether KIAP4 is important for cell adhesion. We generated a KIAP4 deletion mutant cell line expressing SMP1::mCh as a flagellar membrane marker (24, 38). Successful KIAP4 gene deletion was confirmed by PCR (*SI Appendix*, Fig. S4*A*), and KIAP4 deletion did not affect promastigote growth (*SI Appendix*, Fig. S4*B*). The cell lines were then allowed to adhere to gridded glass coverslips for 24 h, followed by quantification of cell adhesion (Fig. 4 *A* and *B*). There was a significant reduction in the number of adhered cells in the KIAP4 null mutant compared with the parental cells (Fig. 4 *A* and *B*). To confirm that the loss of adhesion was specific for KIAP4 deletion, we generated an add-back cell line in which an mNG-tagged version of KIAP4 (KIAP4::mNG) was introduced into the deletion mutant. Successful KIAP4::mNG add-back generation and expression were confirmed by PCR and fluorescence microscopy (*SI Appendix*, Fig. S4 *A*, *C*, and *D*). There was a significant increase in adhered parasites in the KIAP4::mNG add-back cell line compared with the KIAP4 null mutant (Fig. 4 *A* and *B*), confirming that KIAP4 is required for in vitro adhesion. We analyzed the KIAP4::mNG signal intensity in the adhered flagellum of in vitro haptomonads from both the add back and endogenously tagged cell lines (*SI Appendix*, Fig. S4*D*). In both the add back cell line (n = 95) and the endogenously tagged cell line (n = 158), KIAP4::mNG signal was seen in every adhered flagellum, with the signal intensity being slightly lower in the add back cell line.

Next, we investigated the effect of KIAP4 deletion on haptomonad adhesion in the sand fly. Female *Lutzomyia longipalpis* were infected with the parental, KIAP4 null mutant, and KIAP4::mNG add-back cells, and their midguts were examined 6 and 9 d postbloodmeal (PBM) (Fig. 4 *C* and *D* and *SI Appendix*, Fig. S4*E*). At these timepoints, the parasites would have migrated anteriorly and colonized the stomodeal valve (13, 14, 39). We observed minimal differences in the infection rates and intensities between the different cell lines, with all establishing heavy late-stage infections on days 6 and 9 PBM (Fig. 4*C* and *SI Appendix*, Fig. S4 *F* and *G* and Movies S2–S4). However, there were large differences in the location of the parasites in the midgut (Fig. 4*D*). While the parental and add-back cell lines colonized the stomodeal valve by day 9 PBM in ~90% and ~60% of the sand flies examined, respectively, the KIAP4 null mutant, despite reaching the cardia region (Movie S3), failed to colonize the valve. To confirm this, we examined the stomodeal valves of the infected sand flies by fluorescence microscopy (*SI Appendix*, Fig. S4*E*), as the three cell lines expressed the flagellar membrane marker, SMP1, endogenously tagged with mCherry. For the parental and add-back cell lines, we observed fluorescence signal accumulation around the stomodeal valve, associated with adhered parasites, whereas for the KIAP4 null mutant, there was no signal accumulation around the valve (*SI Appendix*, Fig. S4*E*). The slightly lower expression of KIAP4:mNG in the add back cell line, alongside the mNG tag, may contribute to the lower valve colonization rate seen in the add-back. Next, we assessed the percentage of mammalian-infective metacyclic forms present at day 9 PBM (*SI Appendix*, Fig. S4*G*). Metacyclics were present in all cell lines at a similar level. These findings show that KIAP4 is essential for colonization of the stomodeal valve and adhesion in vitro, but is not required for metacyclogenesis.

Parasite colonization and adhesion to the stomodeal valve are associated with damage to the cuticular lining of the valve (7, 13, 16, 17, 40). To assess the changes to the valve on infection, we stained the chitin of the stomodeal valve with calcofluor. We observed a clear calcofluor staining of the stomodeal valve in the noninfected midguts, a pattern that was replicated in midguts of sand flies infected with KIAP4 null mutants (Fig. 4*E*). In contrast, infections with the parental or KIAP4::mNG add-back cell lines resulted in the substantial reduction in the calcofluor signal (Fig. 4*E*). Collectively, these findings show that KIAP4 null mutants do not cause detectable damage to the stomodeal valve.

## Discussion

*Leishmania* adhesion to the sand fly stomodeal valve is mediated by a highly modified flagellum containing an adhesion plaque and a dense filamentous network that extends from the plaque and connects to the anterior cell tip (Fig. 1*A*) (19, 21, 22). Here, using TurboID proximity labeling, we have advanced our knowledge of the plaque protein components and have extended the functional dissection of this cytoskeletal structure at the molecular level through the identification of the ARND family of proteins, including KIAP4. Given the ~10 nm labeling radius of TurboID (30, 31), we believe KIAP4 and the other enriched proteins are likely core components of the haptomonad adhesion plaque, though further work will be required to define their specific localization within the plaque structure. KIAP1 and KIAP2 were not enriched in our proteomic analysis. KIAP1 only has a short intracellular domain that is likely important for protein interactions, which may render it inaccessible for biotinylation. In contrast, KIAP2 is a component of the filamentous domain of the adhered flagellum (18, 24) and is therefore spatially distant from KIAP3, so unlikely to be biotinylated.

We have previously shown that haptomonad adhesion requires progressive disassembly of the motile promastigote flagellum into a short and highly modified haptomonad flagellum containing distinct structural elements, including the adhesion plaque (19). Our colocalization analysis revealed that, like KIAP3, KIAP4 is found within the adhesion plaque adjacent to the surface the flagellum is adhered to. Moreover, KIAP4 appeared alongside KIAP3 within flagellum membrane deformations seen at the earliest stages of *Leishmania* adhesion (19) and then accumulated in the expanded region of the haptomonad flagellum. The expression pattern of KIAP4, its localization to the adhesion plaque, and its biotinylation by TurboID-tagged KIAP3 show that KIAP4 is likely a foundational protein for assembling the adhesion complex and potentially interacts with KIAP3.

Intriguingly, six of the 17 proteins we identified that localized strongly to the adhered flagellum were related to KIAP4. While these proteins had a diverse range of sizes, they all contain a predicted p-loop NTPase domain, which our bioinformatics analysis suggested was inactive; hence, we termed these proteins the Adhesion Related NTPase-like domain (ARND) family. It is likely that not all ARND gene family proteins will be directly involved in parasite adhesion, as they were not detected in our proteomics or only had a weak signal in the adhered flagellum. However, the presence of the NTPase-like domain within these proteins indicates that the parasite has found utility for this domain to assemble and sculpt the adhesion complex, and it will likely be important for mediating protein–protein interactions. This has parallels with the calpain-like protein family. This family is expanded in the kinetoplastids and is characterized by the presence of an inactive calpain domain and has multiple roles in maintaining and regulating the cytoskeleton and cell morphogenesis (25–27, 41, 42).

Among proteins in other organisms that contain an NTPase domain, the septins, though not conserved in kinetoplastids, may provide a framework for understanding the functions of KIAP4. Septins are GTP-binding proteins conserved in many eukaryotes, where they control diverse cellular processes by acting as protein scaffolds and/or as diffusion barriers, and can form filaments that manage membrane shape (43–45). While acknowledging that KIAP4 does not contain an active NTPase domain, the protein has a predicted domain structure that has parallels to septin (44). Hence, we speculate that KIAP4 may have filament-forming capabilities, which could sculpt the membrane of the adhered haptomonad flagellum during differentiation. Deciphering the specific function of KIAP4 will be important in future studies.

The ARND gene family is well conserved across the kinetoplastids. A varying number of paralogs were identified across the species examined, except in *Bodo saltans* and *Trypanosoma rangeli*, which had none. *Bodo saltans* is a free-living kinetoplastid that adheres transiently to substrates in the environment via the flagellum. This transient adhesion is not associated with plaque formation (23, 46, 47), explaining the absence of the ARND gene family. Similarly, *Trypanosoma rangeli* adheres transiently to the salivary gland epithelium of *Rhodnius* spp., with carbohydrates and lectins implicated in these interactions (48, 49). This adhesion mechanism is not associated with ultrastructural changes to the flagellum (49) and is therefore unlikely to require the ARND gene family proteins. Thus, possession of the ARND gene family in the kinetoplastid parasites appears to be correlated with stable adhesion to vector surfaces.

Phylogenetic analysis of the ARND gene family revealed ancient gene duplication events within the family. The clades containing multiple orthologous genes from different species suggest that gene duplication events occurred early in the evolution of these parasites, followed by diversification in specific lineages that may reflect the sculpting of the adhesion complex in response to the environment of their different insect vectors. In *L. mexicana*, seven of the 11 ARND gene family proteins, including KIAP4 strongly localized to the adhered flagellum, yet LmxM.32.2970 was not detected in our proteomics, LmxM.32.2900 did not meet our selection criteria, and a further two (LmxM.17.1030, LmxM.32.2905) despite being enriched in our proteomics, had only a weak signal in the adhered flagellum. This suggests there is functional diversity within this gene family; although several members, such as KIAP4, are likely important for adhesion, some might perform other functions. This pattern will likely be repeated across the kinetoplastids, with some ARND gene family proteins required for adhesion and others for different functions.

In our analysis of data from previous proteomic studies of adhered forms or forms associated with adhesion in other kinetoplastids (35–37), we saw a similar pattern with some but not all KIAP4 paralogs upregulated in the adhered form, except in *C. fasciculata,* in which none of the KIAP4 paralogs were upregulated. For *C. fasciculata,* this may reflect that, despite a role in parasite adhesion, the abundance of these proteins does not change between adhered and nonadhered forms, or they have evolved different functions. In *T. brucei*, four of the eight KIAP4 paralogs were upregulated in cells overexpressing RBP6, which forces the developmental progression of procyclics to epimastigotes (35, 50). In the tsetse fly, *T. brucei* epimastigotes adhere to the salivary gland; however, in culture, these RBP6 overexpression cells do not adhere, yet they express the KIAP4 paralogs that are important for adhesion in *Leishmania*. This raises the possibility that these cells are competent to adhere if presented with a suitable substrate.

The expression level and localization for all but one of these KIAP4 paralogs in the nonadhered *T. brucei* procyclic form are consistent with a role in adhesion, as the signal is weak or cytoplasmic [TrypTag, (51)], suggesting that these proteins are either not expressed in this form or are primed to initiate adhesion. The exception is Tb927.2.5610 (POMP22B), which has been detected in an outer mitochondrial proteome and in TrypTag has a clear mitochondrial localization (51, 52), which may indicate this protein has acquired additional functions in this nonadhered form. In *T. congolense,* two of the nine KIAP4 paralogs were upregulated in the adhered epimastigote form (36). Crucially, we demonstrated that three of these paralogs localized to the lateral side of the flagellum directly in contact with the surface. Together, the upregulation of the *T. congolense* and *T. brucei* KIAP4 paralogs in the epimastigote forms, and the localization of the *T. congolense* KIAP4 paralogs to the adhered region of the flagellum, suggest that the ARND gene family is important for the adhesion of other kinetoplastids to their insect vectors.

We analyzed the function of KIAP4 in *Leishmania*, as this is currently the most tractable organism in which to do these analyses. Deletion of KIAP4 severely impaired *Leishmania* adhesion in vitro. In the sand fly, KIAP4 null mutants were able to proliferate, migrate to the anterior midgut, and undergo metacyclogenesis; however, they failed to colonize the stomodeal valve. Recent single-cell sequencing work showed that late-stage development of *Leishmania* in the sand fly is characterized by a bifurcation with parasites either differentiating to metacyclics or haptomonads (14). The ability of KIAP4 null mutants to proliferate, migrate, and differentiate into metacyclics indicates that the observed adhesion defect in KIAP4 null mutants are related to problems with the generation of haptomonads rather than a general reduction in fitness. As KIAP4 is positioned in the adhesion plaque, the loss of adhesion in KIAP4 deletion mutants likely suggests a disruption to adhesion plaque assembly, resulting in a weakened connection to the underlying substratum.

Stable haptomonad adhesion to the sand fly stomodeal valve is associated with damage to the cuticular lining of the valve that is thought to enhance *Leishmania* transmission through parasite regurgitation into the host (7, 13, 15–17). Additionally, Catta-Preta and colleagues showed that detached haptomonads are infective in mice and exacerbate pathology when co-inoculated with low doses of metacyclics (14). Here, we saw that in sand flies infected with the KIAP4 null mutant, there was no difference in the chitin staining of the stomodeal valve in comparison to the uninfected sand flies. We hypothesize that, given the lack of damage to the stomodeal valve and loss of parasite colonization of the valve, the KIAP4 null mutants would have reduced capacity for transmission and reduced disease severity in the mammalian host.

In conclusion, we have identified a component of the adhesion plaque, KIAP4, which is necessary for *Leishmania* adhesion to the sand fly stomodeal valve, a likely important prerequisite for transmission of the infection to a mammalian host. KIAP4 is the founding member of a set of related proteins, the ARND gene family, that is found in nearly all kinetoplastids. Upregulation of ARND gene family paralogs in *T. brucei* and *T. congolense* epimastigotes and localization of *T. congolense* paralogs to the adhered epimastigote flagellum suggest a common mechanism of adhesion across important pathogenic kinetoplastids, opening avenues for developing transmission-blocking strategies.

## Methods

### Cell Culture.

*Leishmania mexicana* (WHO strain MNYC/BZ/1962/M379) promastigotes expressing Cas9 nuclease and T7 RNA polymerase (C9T7) (32) were grown at 28 °C in M199 medium (Life Technologies) with Earle’s salts, L-glutamine, 10% fetal bovine serum (FBS), 40 mM HEPES-HCl (pH 7.4), 26 mM NaHCO_3_, and 5 µg/mL hemin. *Trypanosoma congolense*, procyclic forms (PCF) were grown at 28 °C in TcPCF-3 medium containing 9.7 g/L MEM Eagle’s powder (Sigma-Aldrich), 26 mM NaHCO_3_, 25 mM HEPES-HCl (pH 7.4), 0.1 mM Hypoxanthine, 10 mM proline, 2 mM glutamine, and 20% FBS. Differentiation to epimastigote forms was performed according to Coustou et al. (53), using starvation medium, TcEMF-1, containing 9.7 g/L MEM Eagle’s powder, 10 mM proline, and 2 mM glutamine. Adherent cells were maintained by replacing the culture supernatant with fresh TcEMF-2 medium containing 9.7 g/L MEM Eagle’s powder, 10 mM proline and 2 mM glutamine and 10% FBS. Additional information is provided in *SI Appendix*, *Material and Methods*.

### Generation of Tagging, Deletion, and Add-Back Cell Lines.

Primers and sgRNAs for the generation of expression and repair cassettes were designed using LeishGEdit (32). Constructs and sgRNA templates for endogenous tagging and deletion were generated by the PCR method according to (32), using pPLOT, pLPOT, or pT as the template. For the KIAP4 add-back construct, the KIAP4 open reading frame was amplified by PCR and cloned into the pJ1364 constitutive expression plasmid (38, 54). The PCR-generated constructs and linearized KIAP4 add-back plasmid were ethanol precipitated. N-terminal eYFP tagging of ARND gene family paralogs in *T. congolense* used the long primer PCR method (55), with pPOTv4 as the template. The PCR-generated plasmid construct was purified using phenol-chloroform (56). Cells were transfected using an Amaxa Nucleofector II. Additional information is provided in *SI Appendix*, *Material and Methods*.

### In Vitro Haptomonad Cell Differentiation.

In vitro haptomonads were generated as described (18, 19, 24). Gridded glass coverslips (iBidi) or scratched Thermanox plastic coverslips (ThermoFisher) were sterilized and washed with M199 and incubated with promastigotes at 28 °C with 5% CO_2_. Biotinylation was initiated in the final 18 h of differentiation by incubating the cells with 50 µM biotin in fresh M199 medium. Additional information is provided in *SI Appendix*, *Material and Methods*.

### TurboID Proteomics.

Coverslips containing adhered cells were washed with Voorheis’s modified PBS (57), then lysed in lysis buffer (1% SDS, 50 mM Tris-HCl, 125 mM NaCl, 1 mM EDTA, 2 mM EGTA, pH 7.4) containing 1 mM PMSF (Sigma-Aldrich), 5 mM Protease Inhibitor Cocktail EDTA-free (Abcam), 2.5x cOmplete protease inhibitor cocktail EDTA-free (Roche), 5 mM 1,10 phenanthroline (Sigma-Aldrich; 131377), 100 µM TLCK (Sigma-Aldrich), and 250U of benzonase endonuclease (Millipore). For the affinity purification and enrichment of biotinylated proteins, the cell lysate was incubated with Sera-Mag streptavidin-coated magnetic beads (Cytiva) at 4 °C overnight. Magnetic beads were washed with a series of buffers.

On-bead digestion of the protein samples was conducted as previously described (58). Beads were resuspended in 50 mM TEAB containing 0.01% ProteaseMAX surfactant (Promega), 10 mM Iodoacetamide, 10 mM tris(2-carboxyethyl) phosphine, and 500 ng Trypsin Lys-C, followed by overnight on-bead digestion at 37 °C. Supernatant from the on-bead digestion was retained, and the beads rinsed with water (LC–MS grade), which was added to the supernatant. The samples were acidified to a final concentration of 0.5% trifluoroacetic acid, followed by centrifugation to pellet insoluble material and degraded ProteaseMax.

Supernatant containing peptides was loaded onto EvoTip Pure tips for desalting and as a disposable trap column for nanoUPLC using an EvoSep One system. A preset EvoSep 100 SPD gradient was used with an EvoSep C_18_ Performance column. The nanoUPLC system was interfaced to a timsTOF HT (Bruker) mass spectrometer with a CaptiveSpray ionization source. Positive PASEF-DIA, nanoESI-MS, and MS^2^ spectra were acquired using Compass HyStar software (v6.3). Data-independent acquisition was performed with 25 Th width windows between 400 to 1,201 Th.

The acquired data in Bruker.d format were searched in Spectronaut (v19.6) against the *Leishmania mexicana* (LmxMC9T7) protein sequence database, appended with common proteomic contaminants. Identifications required precursor Qvalue <0.01 and protein Qvalue <0.01. The resulting protein level data were further filtered to require a minimum of two peptides per accepted protein. Pair-wise statistical testing between sample groups was performed using limma via FragPipe-Analyst (28, 29, 59), run as a local installation in R-Shiny. A minimum imputation was applied to missing values, and the Benjamini–Hochberg approach was used for the multiple test correction. A significance threshold of adjusted *P*-value < 0.05 was applied. Additional information is provided in *SI Appendix*, *Material and Methods*.

### Microscopy.

For live cell microscopy of *Leishmania* cells, log phase cells were washed and incubated with 1 µg/mL of Hoechst 33342. Cells were imaged on a Zeiss Axio ImagerZ2 using the Zeiss 63x/1.4NA PH3 oil-immersion Plan-Apochromat objective and Hamamatsu Flash 4 camera (Hamamatsu). Z-stacks were acquired in ZEN 3.10 Blue software.

To determine TurboID::KIAP3 activity, adhered in vitro haptomonad cells were washed with vPBS and fixed for 10 min at RT with 4% paraformaldehyde, then permeabilized. Cells were blocked for 1 h at RT with 1% BSA in PBS and then incubated for 1 h at RT with streptavidin AlexaFluor 488. Cells were washed, then incubated in PBS with 1 µg/mL of Hoechst 33342. Cells were imaged on a Zeiss ImagerZ2 using the Zeiss 63x/1.4NA PH3 oil-immersion Plan-Apochromat objective and Hamamatsu Flash 4 camera. Z-stacks were acquired with ZEN 3.10 Blue. For all the images, the optimal brightness/contrast adjustments and further analysis were performed using Fiji (60), and the figures were generated using the QuickFigures plugin in Fiji (61).

To determine the localization of ARND gene family paralogs in *T. congolense*, adhered cells were fixed with 3.6% formaldehyde in PBS for 10 min, permeabilized, and incubated with 100 ng /mL DAPI for 3 min. For cytoskeletons, cells were demembranized using 1% IGEPAL CA-630 in PEME for 30 s, fixed for 10 min with 3.6% formaldehyde in PBS, and incubated with 100 ng/mL DAPI. Cells were imaged on a Leica DM5500B microscope controlled by Micromanager, using the 63×/1.4 oil-immersion Plan-Apochromat PH3 objective and the Andor Neo 5.5 sCMOS camera.

For time lapse observation, log phase promastigotes were grown in glass bottom culture dishes (iBidi) for 12 h at 28 °C with 5% CO_2_. Cells about to adhere to the glass were recorded with a Zeiss LSM 880 using the Plan-Apochromat 63×/1.4 oil DIC M27 objective. During imaging, cells were incubated at 28 °C with 5% CO_2_.

For confocal microscopy, log phase promastigotes were cultured in glass bottom dishes (iBidi) for 24 h at 28 °C with 5% CO_2_. The dish was washed with M199 to remove nonadherent cells. Confocal microscopy was performed in superresolution mode using a Zeiss LSM880, with Airyscan detector, and a Zeiss Plan-Apochromat 100×/1.46 Oil DIC M27 Elyra objective. Images were Airyscan processed using the default parameters with ZEN 2.3 SP1 FP3 Black (v14.0). Further analyses were performed using Fiji (60). Additional information is provided in *SI Appendix*, *Material and Methods*.

### Quantification of Adhered In Vitro Haptomonad Cells.

Log phase promastigotes were grown on gridded coverslips in M199 for 24 h at 28 °C with 5% CO_2._ The coverslips were washed, then incubated for 5 min in DMEM with Hoechst 33342. Adhered cells were imaged on a Zeiss Axio ImagerZ2 using the Zeiss Plan-Apochromat 20x/0.8 PH2 objective and Hamamatsu Flash 4 camera. For each coverslip, images of adhered cells in five different grid areas were acquired with ZEN Blue (v3.10). Additional information is provided in *SI Appendix*, *Material and Methods*.

### Sand Fly Infections.

Female *Lutzomyia longipalpis* sand flies were fed through a chick-skin membrane (62) on heat-inactivated sheep blood spiked with 1 × 10^6^ cells/mL of log-phase promastigotes. Blood-fed females were isolated and maintained at 26 °C with access to a 50% sugar solution. On days 6 and 9 post bloodmeal, sand flies were dissected and digestive tracts examined by light microscopy. *Leishmania* infection intensities were graded as negative, light (<100 parasites/gut), moderate (100 to 1,000 parasites/gut), and heavy (>1,000 parasites/gut). All the infection experiments were performed in two independent replicates. For the calcofluor assay, midguts dissected in saline were incubated with Calcofluor White Stain (Sigma-Aldrich) for 1 min, then washed with saline. Samples were imaged using an Olympus BX51 microscope. Additional information is provided in *SI Appendix*, *Material and Methods*.

### Bioinformatics and Phylogenetic Analyses.

To identify the complete ARND family in *L. mexicana*, the KIAP4 (LmxM.32.2940) protein sequence was used to perform a BLASTP search against the *L. mexicana* proteome with an e-value threshold of 10^−5^ (63). Each of the identified proteins was used as the query in an iterative BLASTP search. Domain architecture prediction was conducted using InterPro (v106.0). Additional p-loop NTPase domains were identified using Foldseek. Prediction of the coiled-coil regions was performed with DeepCoil2 (64–66), while intrinsically disordered regions were predicted with the MobiDB-lite. Conservation of the Walker A motif in the ARND gene family was determined by searching the AlphaFold3 predicted protein structures of each of the ARND gene family members using Foldseek in 3Di/AA mode to identify the best match for each p-loop NTPase domain from a common model organism (67, 68). The Walker A motif from the identified p-loop NTPase domain was mapped onto the ARND gene family protein sequence.

To determine the presence of the ARND gene family in other kinetoplastids, we performed an exhaustive iterative BLASTP search against the proteome of other kinetoplastids using the 11 *L. mexicana* proteins. Identified proteins were then used as the query in a second round of exhaustive iterative BLASTP search against the proteome of that kinetoplastid.

To infer evolutionary conservation of the ARND family, we analyzed ARND gene family protein sequences from *Leishmania mexicana, Crithidia fasciculata, Paratrypanosoma confusum, Trypanosoma brucei, Trypanosoma congolense,* and *Trypanosoma cruzi*, representing the two morphological superclasses in kinetoplastids (34). Multiple sequence alignment was performed using MAFFT v7.511 (69, 70) with the L-INS-i iterative refinement method and default parameters. A maximum likelihood tree was inferred with the IQ tree (v1.6.12) (71) using the VT+F + I + G4 substitution model selected under the Bayesian information criterion with the built-in ModelFinder (72). Branch supports were evaluated by ultrafast bootstrap analysis based on 1,000 replicates (73). The tree topology was further evaluated, over 1,000 bootstrap replicates, with PhyML v3.0 using the maximum likelihood method and Q.pfam +R + F substitution model (74, 75). Statistically unsupported branches with bootstrap values <70% were collapsed. Additional information is provided in *SI Appendix*, *Material and Methods*.

### Statistical Analysis.

Descriptive statistics, including protein counts and means, were determined using Microsoft Excel. Where appropriate, the number of samples or technical replicates analyzed is given in the text or figure legends. Statistical comparison between groups was estimated using the two-tailed Welch’s *t* test in GraphPad Prism (v9.5) at a *P* < 0.05 significance level. Data were plotted using GraphPad Prism.

## Supplementary Material

Appendix 01 (PDF)

Dataset S01 (XLSX)

Dataset S02 (XLSX)

Dataset S03 (PDF)

Movie S1. **Time-lapse movie showing the accumulation of KIAP4::mNG signal in the expanded region of the flagellum during adhesion of in vitro haptomonad cells**. The timestamp in hours: minutes: seconds is shown at the top right of the movie for a playback of ~8.4 h at 1008× speed.

Movie S2. Real-time observation of the dissected thoracic midgut of the sand fly infected with the *L. mexicana* parental cell line on day 9 post blood meal (PBM).

Movie S3. Real-time observation of the dissected thoracic midgut of the sand fly infected with the *L. mexicana* KIAP4 null mutant cell line on day 9 PBM.

Movie S4. Real-time observation of the dissected thoracic midgut of the sand fly infected with the *L. mexicana* KIAP4::mNG add-back cell line on day 9 PBM.

## Data Availability

Mass spectrometry proteomics datasets generated in this work have been deposited in the ProteomeXchange Consortium (76) via the PRIDE (77) partner repository with the dataset identifier PXD072873, and the MassIVE under accession code MSV000100393. All data reported in this work, and any additional information required to reanalyze the data, will be shared by the lead contact upon request.
