# Supplementary material for: Identification of a conserved gene family with an essential role in Leishmania parasite–insect vector adhesion
Source: Proc Natl Acad Sci U S A. 2026 Jul 20;123(30):e2603653123. doi: 10.1073/pnas.2603653123 (PMC13416946; doi:10.1073/pnas.2603653123)
Supplement: Supplementary file 1 — Appendix 01 (PDF) [file pnas.2603653123.sapp.pdf]

PNAS

Supporting Information for

Identification of a conserved gene family with an essential role in *Leishmania* parasite – insect vector adhesion

Barrack O Owino, Ryuji Yanase, Katerina Pruzinova, Helen Farr, Yaimie Lopez, Alan O Marron, Sue Vaughan, Petr Volf, Jack D Sunter

Corresponding authors: Jack D Sunter; Petr Volf
Email: jsunter@brookes.ac.uk; volf@cesnet.cz

This PDF file includes:

SI Material and Methods
Figures S1 to S4
Tables S1
Legends for Movies S1 to S4
Legends for Datasets S1 to S3
SI References

Other supporting materials for this manuscript include the following:

Movies S1 to S4
Datasets S1 to S3

SI Material and Methods

Cell culture

Leishmania mexicana (WHO strain MNYC/BZ/1962/M379) promastigotes expressing Cas9 nuclease and T7 RNA polymerase (C9T7) [1] were grown at 28°C in M199 medium (Life Technologies) with Earle's salts, L-glutamine, 10% foetal bovine serum (FBS), 40 mM HEPES-HCl (pH 7.4), 26 mM $NaHCO_3$ and 5 µg/ml haemin. Cells were maintained in logarithmic growth by regular subculturing.

Trypanosoma congolense, procyclic forms (PCF) were grown at 28°C in TcPCF-3 medium containing 9.7 g/L MEM Eagle's powder (Sigma; M0643), 26 mM $NaHCO_3$, 25 mM HEPES-HCl (pH 7.4), 0.1 mM Hypoxanthine, 10 mM proline, 2 mM glutamine and 20% foetal calf serum (FCS). Cells were maintained in logarithmic growth by changing the medium every 3 days. Induction of differentiation to epimastigote forms (EMF) was performed according to Coustou *et al* [2], using a starvation medium, TcEMF-1, containing 9.7 g/L MEM Eagle's powder, 10 mM proline and 2 mM glutamine. $5x10^7$ PCF cells were pelleted by centrifugation at 1600x g for 10 min, resuspended in 5 ml of TcEMF-1 medium, and incubated at 28°C for several hours before the addition of 10% FCS. Adherent EMF cells were maintained in culture by replacing the culture supernatant with fresh TcEMF-2 medium containing 9.7 g/L MEM Eagle's powder, 10 mM proline and 2 mM glutamine and 10% FCS.

Growth curve

Leishmania mexicana promastigotes at a density of 1 x 10^5 cells/ ml were grown in M199 supplemented with 10% FBS. Daily cumulative cell growth was determined using a Beckman Z2 Coulter Counter.

Generation of tagging, deletion and add-back cell lines

Primers and sgRNAs for the generation of expression and repair cassettes were designed using LeishGEdit (http://www.leishGEdit.net) [1] (Table S1). Tagging constructs and sgRNA templates for endogenous mCherry (mCh), mNeonGreen (mNG) or TurboID tagging were generated by the PCR method according to Dean *et al* [3] and Beneke *et al* [1], using pPLOT or pLPOT plasmids with either Blasticidin, neomycin, or Puromycin resistance genes as the templates.

The KIAP4 (*LmxM.32.2940*) gene deletion construct was generated by PCR using pTPuro and pTBlast plasmids as templates and the G00 primer [1]. For the KIAP4 add-back construct, the KIAP4 open reading frame was amplified by PCR (Table S1) and cloned into the SpeI and HindIII restriction sites of the pJ1364 constitutive expression plasmid, with Phleomycin resistance gene, to generate a C-terminally tagged version of KIAP4 with an mNG fused to a triple myc tag [4,5]. 10 µg of the plasmid construct was linearised by digestion with PacI (New England Biolabs, Ipswich, UK). The PCR-generated constructs and linearised KIAP4 add-back plasmid were precipitated with 100% ethanol and 3M sodium acetate (pH 5.2) and transfected using the X-001 programme on an Amaxa Nucleofector II. Successful transfectants were selected after 6 hours using Puromycin (20 µg/ml), G-418 (20 µg/ml), Blasticidin (5 µg/ml) or Phleomycin (25 µg/ml) (Melford Laboratories).

N-terminal eYFP tagging of the three ARND gene family paralogs in *T. congolense* was based on the long primer PCR method [3], using the pPOTv4 plasmid with Blasticidin resistance gene as the template (Table S1). The PCR-generated plasmid construct was purified using phenol-chloroform [6] before transfection. Cells were transfected using an Amaxa Nucleofector II, and successful transfectants were selected using 5 µg/ml Blasticidin (Melford Laboratories).

In vitro haptomonad cell differentiation

Axenic haptomonad cells were generated as described previously [7–9]. Gridded glass coverslips grid-500 (iBidi; 10816) were cut into small pieces of ~5 × 5 mm and sterilised with 100% ethanol for 5 min. The coverslips were washed twice with 5 mL M199 and transferred into a 24-well plate containing 1 mL of M199. Coverslips were washed two more times with 1 mL of M199 and incubated with 5 × 10^6 cells/ mL promastigotes at 28°C with 5% CO_2 for 24 h (for localisation analysis) or 72 h, with the medium being changed every 24 h (for TurboID::KIAP3 activity determination in adhered cells). For the latter experiment, biotinylation was initiated in the final 18 h of in vitro haptomonad cell differentiation by incubating the cells in fresh M199 medium supplemented with 50 µM biotin.

For the proteomics, 72 Thermanox plastic coverslips (ThermoFisher; 174950) per cell line were scratched uniformly using polishing paper, sterilised with 100% ethanol, and

washed as described above. Parental and TurboID::KIAP3 promastigotes at 5 x 10^6 cells/ mL were allowed to adhere on the coverslips in a 24-well plate with 1 mL of M199 medium for 72 h at 28°C with 5% CO_2, with the medium being replaced every 24 h. In the final 18 h of in vitro haptomonad cell differentiation, biotinylation was initiated by incubating the cells in fresh M199 medium supplemented with 50 µM biotin.

TurboID-tagged KIAP3 proteomics

After 72 hours of in vitro haptomonad differentiation and biotinylation, the coverslips containing adhered cells from the parental and TurboID::KIAP3 cell lines were transferred to a fresh 24-well plate and washed three times with 1 ml of pre-warmed Voorheis's modified PBS (vPBS; 3 mM KCl, 16 mM Na_2HPO_4, 137 mM NaCl, 3 mM KH_2PO_4, 46mM sucrose, 10mM glucose, pH 7.6) [10]. Adhered cells from each cell line were lysed in 1200 µl lysis buffer (1% SDS, 50 mM Tris-HCl, 125 mM NaCl, 1 mM EDTA, 2mM EGTA, pH 7.4) containing 1 mM PMSF (Sigma-Aldrich; 7110-OP), 5 mM Protease Inhibitor Cocktail EDTA-free (Abcam; ab201111), 2.5x cOmplete protease inhibitor cocktail EDTA-free (Roche; 4693159001), 5 mM 1,10 phenanthroline (Sigma-Aldrich; 131377), 100 µM TLCK (Sigma-Aldrich; 616382) and 250U of benzonase endonuclease (Millipore; E1014). 20 µl aliquots of the lysate from each cell line were saved as input material, while the rest were subjected to affinity purification. For the affinity purification and enrichment of biotinylated proteins, 1100 µL of the lysate from each cell line was incubated with 135 µl of Sera-Mag streptavidin-coated magnetic beads (Cytiva; 30152105011150) by end-over-end rotation at 4ºC overnight. Magnetic beads were collected using a magnetic rack, and 20 µL aliquots of the unbound material (flow-through) were used for Western blotting. Beads were washed four times with 500 µl of the lysis buffer for 5 min each, once with 500 µl of 4 M urea in 50 mM triethylammonium bicarbonate (TEAB; pH 8.5) for 2 min, once with 500 µl of 6 M urea in TEAB for 2 min, once with 500 µl of 1 M KCl for 5 min, and once with 500 µl of TEAB for 5 min. For each cell line,15 µL aliquots of the beads from the last wash were resuspended in 1x sample loading buffer (2% SDS, 50 mM DTT, 60 mM Tris-HCl, 1x cOmplete protease inhibitor cocktail) containing 5 mM biotin and eluted by boiling at 95ºC for 5 min [11]. The input, flow-through and eluate materials were analysed by Western blotting to check for biotinylated proteins and TurboID::KIAP3 expression before on-bead digestion of biotinylated proteins and mass spectrometry.

LC-MS/MS data acquisition

On-bead digestion of the protein samples from three technical replicates was conducted as previously described [12]. Beads from each cell line were resuspended in 200 µl of 50 mM TEAB containing 0.01% ProteaseMAX surfactant (Promega), 10 mM Iodoacetamide (IAM), 10 mM tris(2-carboxyethyl) phosphine (TCEP), and 500 ng Trypsin Lys-C, followed by overnight on-bead digestion at 37ºC. Supernatant from the on-bead digestion was retained, and the beads rinsed for 5 min with 50 µl of water (LC-MS grade), which was then added to the supernatant. The samples were acidified to a final concentration of 0.5% trifluoroacetic acid (TFA), followed by centrifugation at 1700xg for 10 mins to pellet insoluble material and degraded ProteaseMax.

Supernatant containing peptides (60 µL) was loaded onto EvoTip Pure tips for desalting and as a disposable trap column for nanoUPLC using an EvoSep One system. A pre-set EvoSep 100 SPD gradient was used with an 8 cm EvoSep C_{18} Performance column (8 cm x 150 µm x 1.5 µm). The nanoUPLC system was interfaced to a timsTOF HT (Bruker) mass spectrometer with a CaptiveSpray ionisation source. Positive PASEF-DIA, nanoESI-MS and MS^2 spectra were acquired using Compass HyStar software (version 6.3, Bruker). Data independent acquisition (DIA) was performed with 25 Th width windows between 400-1201 Th. Instrument source settings were: capillary voltage, 1600 V; dry gas, 3 L/min; dry temperature, 180°C. TIMS settings were: 1/K0 0.6–1.6 V.s/cm^2; ramp time, 100 ms; ramp rate, 9.42 Hz; collision energy was interpolated between 20 eV at 0.6 V.s/cm^2 – 59 eV at 1.6 V.s/cm^2.

LC-MS/MS data analysis

The acquired data in Bruker.d format were searched in Spectronaut (v19.6) against the *Leishmania mexicana* (LmxMC9T7) protein sequence database, appended with common proteomic contaminants. The search parameters included specific Trypsin/P, LysC/P (C-terminal KR) enzymes, carbamidomethyl (C) as a fixed modification, and Oxidation (M), and Acetyl (protein N-term) as variable modifications. Identifications required precursor Qvalue <0.01 and protein Qvalue (Experiment) <0.01. The resulting protein level data were further filtered to require a minimum of two peptides per accepted protein. Pair-wise statistical testing between sample groups was performed using limma via FragPipe-Analyst [13–15], run as a local installation in R-Shiny. A minimum imputation was applied to missing values, and the Benjamini-Hochberg approach was

used for the multiple test correction. A significance threshold of adjusted p-value <0.05 was applied. Three independent in vitro haptomonad differentiations with biotinylation were performed and analysed. The first replicate was performed at a different time from the subsequent two replicates, which were performed in parallel. In our analysis, we either analysed all three replicates or the two replicates performed in parallel (Supplementary Data 1). There was good reproducibility with 71 of the top 100 enriched proteins common between the analysis of the two parallel replicates and all three replicates.

Widefield microscopy

For live cell microscopy of the tagged promastigote cells, 1 mL (~$1x10^7$ cells) of log phase cell cultures was washed twice with 1 mL of DMEM, incubated for 5 min at RT in DMEM containing 1 μg/mL of Hoechst 33342 and washed twice with 1 mL DMEM. Cells were resuspended in 100 μl of DMEM, and 1 μL of the suspension was added to the centre of premarked circular wells on a Super Frost microscope slide and mounted with a glass coverslip. Cells were imaged on an upright Zeiss Axio ImagerZ2 microscope using the Zeiss 63x/1.4NA PH3 oil-immersion Plan-Apochromat objective and Hamamatsu Flash 4 camera (Hamamatsu Photonics, Hamamatsu, Japan).

For the in vitro haptomonad cell imaging, cells adhered on ~5 × 5 mm gridded glass coverslip were washed twice in 1 mL of DMEM and incubated in DMEM with 1 μg/mL of Hoechst 33342 for 5 min. Coverslips were washed twice with 1 mL DMEM and mounted onto a Super Frost microscope slide with the side containing adhered cells facing up. Another coverslip was mounted on top, followed by imaging using the Zeiss ImagerZ2 microscope with the Zeiss 63x/1.4NA PH3 oil-immersion Plan-Apochromat objective and Hamamatsu Flash 4 camera. Z-stacks were acquired in ZEN 3.10 Blue software using the phase contrast (TL lamp illumination at 35.6% power and 5 ms exposure time), mCherry (Zeiss filter set 43 HE, 150 ms exposure, and Colibri7 LED at 590 nm wavelength and 20% power), EGFP (Zeiss filter set 38 HE, 150 ms exposure, and Colibri7 LED at 475 nm wavelength and 10% power) and H33342 (Zeiss filter set 49, 40 ms exposure time, and Colibri7 LED at 385 nm wavelength and 20% power) channels, and a voxel size of 0.103 μm x 0.103 μm x 0.25 μm. The strength of the fluorescence signal in the adhered flagellum was determined qualitatively based on the presence of a strong, weak, or no signal in the adhered flagellum, using the signal intensity of the

haptomonad cell as the baseline reference. Quantitation of the KIAP4::mNG signal was measured using Fiji. The area of the plaque was defined, and the average signal intensity was measured before subtraction of the background.

To determine TurboID::KIAP3 biotin ligase activity in adhered in vitro haptomonad cells, coverslips in which cells had been allowed to adhere for 72 h were washed three times with pre-warmed vPBS and fixed for 10 min at RT with ice-cold 4% paraformaldehyde (PFA) in vPBS. Unreacted PFA was quenched for 5 min at RT with 1% glycine in PBS. Cells were permeabilised with 0.1% IGEPAL CA-630 (Sigma; I3021) in PEME for 5 min, washed twice with ice-cold PBS for 2 min each, and blocked for 1 h at RT with 1% BSA in PBS. Cells were then incubated for 1 h at RT with streptavidin AlexaFluor 488 diluted at 1:200 in the blocking buffer, washed four times with PBS for 5 min each, and incubated in PBS with 1 μg/mL of Hoechst 33342 for 5 min. The cells were washed once in PBS for 5 min and mounted with Vectashield mounting medium for imaging on the Zeiss ImagerZ2 microscope using the Zeiss 63x/1.4NA PH3 oil-immersion Plan-Apochromat objective and Hamamatsu Flash 4 camera. Z-stacks were acquired with the ZEN 3.10 Blue software on the phase contrast (5 ms), EGFP (150 ms) and H33342 (40 ms) channels, and a voxel size of 0.103 μm x 0.103 μm x 0.25 μm. For all the images, the optimal brightness/contrast adjustments and further analysis were performed using Fiji [16], and the figures were generated using the QuickFigures plugin in Fiji [17].
To determine the localisation of ARND gene family paralogs in adhered *T. congolense* epimastigote forms (EMF), procyclic forms (PCF) were induced to differentiate to EMF directly onto coverslips in TcEMF media. This allowed imaging of the entire adhered flagellum. Adhered EMF cells were fixed with 3.6% formaldehyde in PBS for 10 min, permeabilised with 0.1% IGEPAL CA-630 (Sigma; I3021) in PEME for 5 min, and incubated with 100 ng /ml DAPI for 3 min. Cells were washed after each step with PBS and mounted for imaging. For the adhered EMF cytoskeleton preparation, cells were demembranised using 1% IGEPAL CA-630 in PEME for 30 sec, fixed for 10 min with 3.6% formaldehyde in PBS, and incubated with 100 ng /ml DAPI for 3 min. Cells were washed as before and mounted for imaging on an upright Leica DM5500B (Milton Keynes, UK) microscope with an Osram HBO mercury short arc 103 W/2 bulb, using the 63×/1.4 oil-immersion Plan-Apochromat PH3 objective and the Andor Neo 5.5 sCMOS camera (Belfast, UK). The images were acquired with the Micromanager (no single version) software using the phase contrast (TL-PH illumination, 3 ms exposure time),

eYFP (YFP filter cube, 500/20 nm excitation, 515 nm dichroic, and 535/30 nm emission, 2000 ms exposure) and DAPI (A4 filter cube, 360/40 nm excitation, 400 nm dichroic, and 470/40 nm emission, 15 ms exposure) channels.

Time-lapse observation

Log phase promastigotes (1×10^6 cells/ml) were grown in 2 ml of M199 in the iBidi's µ-dish 35 mm, high glass bottom (Gräfelfing, Germany) culture dishes for 12 hr at 28°C with 5% CO_2. The dishes were washed five times with fresh M199 medium before imaging. Cells about to adhere to the glass were recorded with an inverted Zeiss LSM 880 (Carl Zeiss, Jena, Germany) using the Plan-Apochromat 63×/1.4 oil DIC M27 objective. Videographs of cells within a 512 x 512 frame size were recorded at 8-bit depth using a bi-directional scan, 100 µm pinhole size, 488 nm excitation and 493-598 nm emission wavelengths for mNG, GaAsP-PMT detector at 830 gain, and argon laser at 0.5% laser power. The transmitted light was recorded using the T-PMT detector at 400 gain. During imaging, cells were incubated at 28°C with 5% CO_2.

Confocal microscopy

Log phase promastigotes (1×10^6 cells/ml) were cultured in 2 mL of M199 in a µ-dish 35 mm (high glass bottom) culture dish for 24 h at 28°C with 5% CO_2. The dish was washed with 2 mL of fresh M199 medium more than five times to remove non-adherent cells before imaging. Confocal microscopy was performed in super-resolution mode using an inverted Zeiss LSM880 with Airyscan detector, a Zeiss Plan-Apochromat 100×/1.46 Oil DIC M27 Elyra objective, and the AxioObserver.Z1 stage. An excitation wavelength of 488 nm and 561 nm was used to observe mNeonGreen and mCherry, respectively, using 2% laser powers and 800 gain. 272 x 272 z-stack images of adhered cells were acquired at 16-bit depth using 2-line averaging, bi-directional scanning, 40 nm × 40 nm x 100 nm voxel size, 0.66 µs/pixel dwell time, and Airyscan processed using the default parameters with ZEN 2.3 SP1 FP3 Black (release version 14.0) software. Further analyses were performed using Fiji [16].

Quantification of adhered in vitro haptomonad cells

Three pieces of ~5 × 5 mm gridded glass coverslips grid-500 (iBidi; 10816) were prepared as described above for each cell line. Log phase promastigotes (5×10^6

cells/ml) of KIAP4 parental, knockout, and add-back cell lines were grown on the coverslips in a 24-well plate with 1 ml of M199 medium for 24 hr at 28°C with 5% CO_2. The coverslips were washed twice by transferring them to wells with 1 ml of DMEM, incubated for 5 min in 1 ml of DMEM with 1 µg/ml of Hoechst 33342, and washed twice with 1 ml of DMEM. The coverslips were mounted on a Super Frost microscope slide, with the top side facing up, and another coverslip was carefully mounted on top to avoid air bubbles. Adhered cells in a 500 µm × 500 µm grid area were imaged on the Zeiss Axio ImagerZ2 upright microscope using the Zeiss Plan-Apochromat 20x/0.8 PH2 objective and Hamamatsu Flash 4 camera. For each coverslip, images of adhered cells in five different grid areas were acquired with ZEN Blue (v3.10) software on the phase contrast (5 ms exposure) and H3342 (50 ms exposure time) channels. The number of adhered cells in each grid area was manually counted in Fiji [16] using the phase contrast image.

Sand fly infections

Female *Lutzomyia longipalpis* sand flies were fed through a chick-skin membrane [18] on heat-inactivated sheep blood spiked with 1 × 10^6 cells/ml of log-phase promastigotes (day 3 – 4) from the parental, KIAP4 null mutant, and KIAP4::mNG add-back cell lines expressing SMP1::mCh as a flagellar membrane marker. Blood-fed females were isolated and maintained at 26°C with free access to a 50% sugar solution. On day 9 post bloodmeal sample, sand flies were dissected and digestive tracts examined by light microscopy. Infections were quantitatively assessed by transferring each gut into 150 µl of 0.01% formaldehyde solution, followed by homogenization and counting using a Burker chamber. *Leishmania* cells with flagellar length >2 times body length were scored as metacyclic form, and those with flagellar length <2 times body length as other promastigote forms [9,19]. Gut smears of *L. mexicana* infected females on day 9 post-infection (PI) were fixed with methanol, stained with Giemsa and examined under the light microscope (Olympus BX51) with an oil-immersion objective. Four morphological forms were distinguished, based on the criteria of Walters [20] and Rogers *et al*. [21]: (i) leptomonads: body length < 14 µm and flagellar length < 2 times body length; (ii) nectomonads: body length ≥ 14 µm; (iii) metacyclic promastigotes: body length < 14 µm and flagellar length ≥ 2 times body length, and (iv) haptomonads: characterised by a reduced flagellum with an enlarged flagellar tip. On 6 and 9 days post-blood meal (PBM), females were dissected in drops of saline solution, and the individual guts were

examined for the presence and localisation of *Leishmania* using an Olympus BX51 microscope with the Olympus UplanApo 20x/0.70 objective and the Olympus DP72 camera. The images were acquired with QuickPHOTO MICRO 3.0 software using phase contrast + Nomarski interference contrast (18 ms exposure time) and mCherry (Olympus U-MWG2 Fluorescence Filter Cube, BP 510-550nm, LP 590nm, 50 ms exposure time) channels. Haptomonads adhere to the sand fly stomodeal valve; therefore, special emphasis was given to the colonisation of the valve. *Leishmania* infection intensities were graded as negative, light (<100 parasites/gut), moderate (100–1000 parasites/gut) and heavy (>1000 parasites/gut). All the infection experiments were performed in two independent replicates.

Calcofluor assay

Midguts dissected in saline were incubated with Calcofluor White Stain (Sigma-Aldrich), which specifically stains chitin. After 1 min of incubation, the calcofluor was replaced with saline. Samples were then examined under UV light using an Olympus BX51 microscope using the Olympus Wide UV-excitation filter, BP 340-390 nm, LP 420 nm, and 33 ms exposure time.

Bioinformatics and Phylogenetic analyses

The sequences of KIAP4 and other ARND gene family proteins identified from the TurboID::KIAP3 proteomics were retrieved from TriTrypDB [22]. To identify other proteins within this family in *Leishmania mexicana*, the KIAP4 (LmxM.32.2940) protein sequence was used to perform an initial BLAST search against the *L. mexicana* (MHOM/GT/2001/U1103) proteome on TriTrypDB using BLASTP with a minimum e-value threshold of 10^{-5} [23]. Each of the identified proteins was then used as the query in an exhaustive iterative BLASTP search against the *L. mexicana* (MHOM/GT/2001/U1103) protein sequence database on TriTrypDB, and all the unique hits satisfying the e-value threshold were combined to determine the number of proteins within this family for *L. mexicana*. Domain architecture prediction was conducted using InterPro (v106.0) via the EMBL-EBI web server (https://www.ebi.ac.uk/interpro/search/sequence/). Additional p-loop NTPase domains were identified using Foldseek (see below). Prediction of the coiled-coil regions was performed with DeepCoil2 using the MPI Bioinformatics toolkit (https://toolkit.tuebingen.mpg.de/tools/deepcoil2) [24–26], while intrinsically disordered

regions were predicted with the MobiDB-lite implemented using the EMBL-EBI's InterPro database. Potential transmembrane domains and signal peptides were predicted using DeepTMHMM server v. 1.0 (https://services.healthtech.dtu.dk/services/DeepTMHMM-1.0/) [27] and SignalP 6.0 (https://services.healthtech.dtu.dk/services/SignalP-6.0/), respectively. Conservation of the Walker A motif in the ARND gene family was determined by searching the AlphaFold3 predicted protein structures for each of the ARND gene family members against seven databases (AlphaFold/Proteome, AlphaFold/Swiss-Prot, AlphaFold/UniProt50, CATH50, GMGCL, MGnify-ESM30, PDB100) using Foldseek in 3Di/AA mode to identify the best match for each p-loop NTPase domain from a common model organism [28,29]. The Walker A motif from the identified p-loop NTPase domain was then mapped onto the ARND gene family protein sequence.

To determine the presence of the ARND gene family in other kinetoplastids, we performed an exhaustive iterative BLASTP search against the proteome of other kinetoplastids that employ different adhesion modes, using the 11 *L. mexicana* proteins within the family as the query. Identified proteins from each kinetoplastid species were then used as the query in a second round of exhaustive iterative BLASTP search against the proteome of the kinetoplastid to identify additional proteins that were potentially missed in the first round. For the kinetoplastid species that were not available on TritrypDB (*Phytomonas serpens* and *Herpetomonas muscarum*), we performed the analyses using the NCBI's tblastn and the Whole-genome shotgun contigs database.
To infer evolutionary conservation of the family across kinetoplastids, we analysed ARND gene family protein sequences from *Leishmania mexicana, Crithidia fasciculata, Paratrypanosoma confusum, Trypanosoma brucei, Trypanosoma congolense,* and *Trypanosoma cruzi*, representing the two morphological superclasses in kinetoplastids [30]. Multiple sequence alignment was performed using MAFFT v7.511 server (https://mafft.cbrc.jp/alignment/server/index.html) [31,32] with the L-INS-i iterative refinement method and default parameters. A maximum likelihood tree was inferred with the IQ tree (v1.6.12) web server (http://iqtree.cibiv.univie.ac.at/) [33] using the VT+F+I+G4 substitution model selected under the Bayesian information criterion with the built-in ModelFinder [34]. Branch supports were evaluated by ultrafast bootstrap analysis based on 1000 replicates [35]. The tree topology was further evaluated, over 1000 bootstrap replicates, with PhyML v3.0 web server (http://www.atgc-montpellier.fr/phyml/)

using the maximum likelihood method and Q.pfam +R+F substitution model, selected under the Bayesian information criterion with the built-in Smart Model Selection [36,37]. Statistically unsupported branches with bootstrap values <70% were collapsed. The resulting gene tree was then used to map the expression patterns of ARND gene family proteins identified with our TurboID::KIAP3 proteomics and protein localisation in *L. mexicana* in vitro haptomonad cells. Genes enriched by at least 2-fold change in *T. brucei* epimastigote form vs. procyclic form after RBP6 overexpression were retrieved from [38], while those for *T. congolense* (IL3000) were identified and retrieved from TriTrypDB [22], using the quantitative mass spec evidence feature. Datasets for *Crithidia fasciculata* adhered vs free swimming forms were obtained from [39]. Protein localisation data for *T. brucei* procyclics were obtained from TrypTag [40].

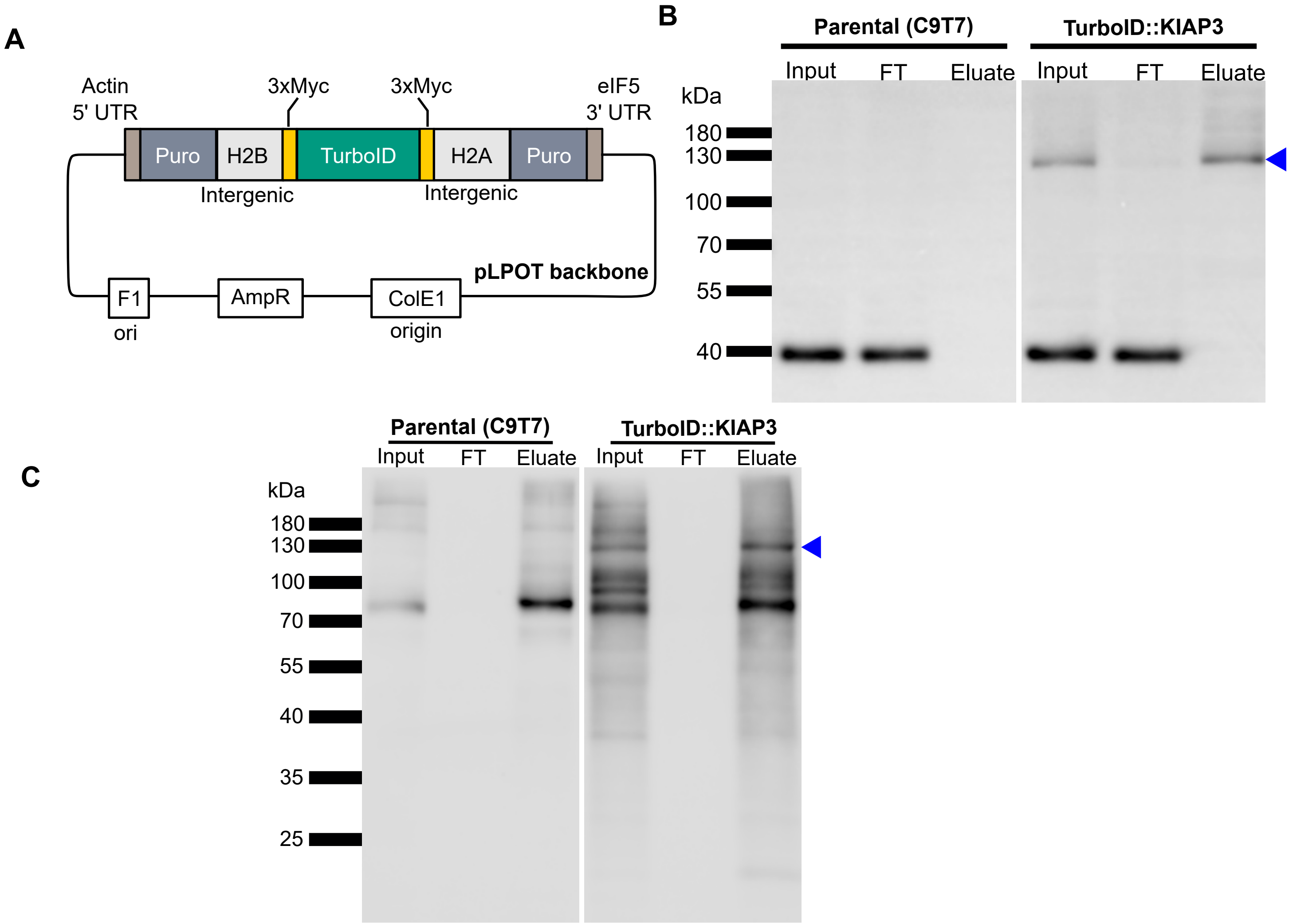

Figure S1. Resolving the adhesion plaque components of in vitro haptomonad cells by TurboID::KIAP3 proximity labelling, Related to Figure 1 and Dataset S1 and S2. (A) Structure of the pLPOT-TurboID plasmid construct used for generating a stable *L. mexicana* cell line expressing KIAP3 endogenously tagged with TurboID at the N-terminus (TurboID::KIAP3). (B and C) Western blots of whole-cell lysates before incubation with streptavidin-coated magnetic beads (input), flow-through (FT) after incubation with the beads, and the eluted materials from the beads (eluate). Samples were probed with anti-Myc antibody, c-Myc Monoclonal Antibody (9E10), to assess TurboID::KIAP3 expression (B) or streptavidin-HRP to evaluate biotinylation (C). The arrowheads (blue) indicate a band corresponding to the position of 3x-Myc-TurboID::KIAP3 (~118 kDa). See also Figure 1 and Dataset S1 and S2.

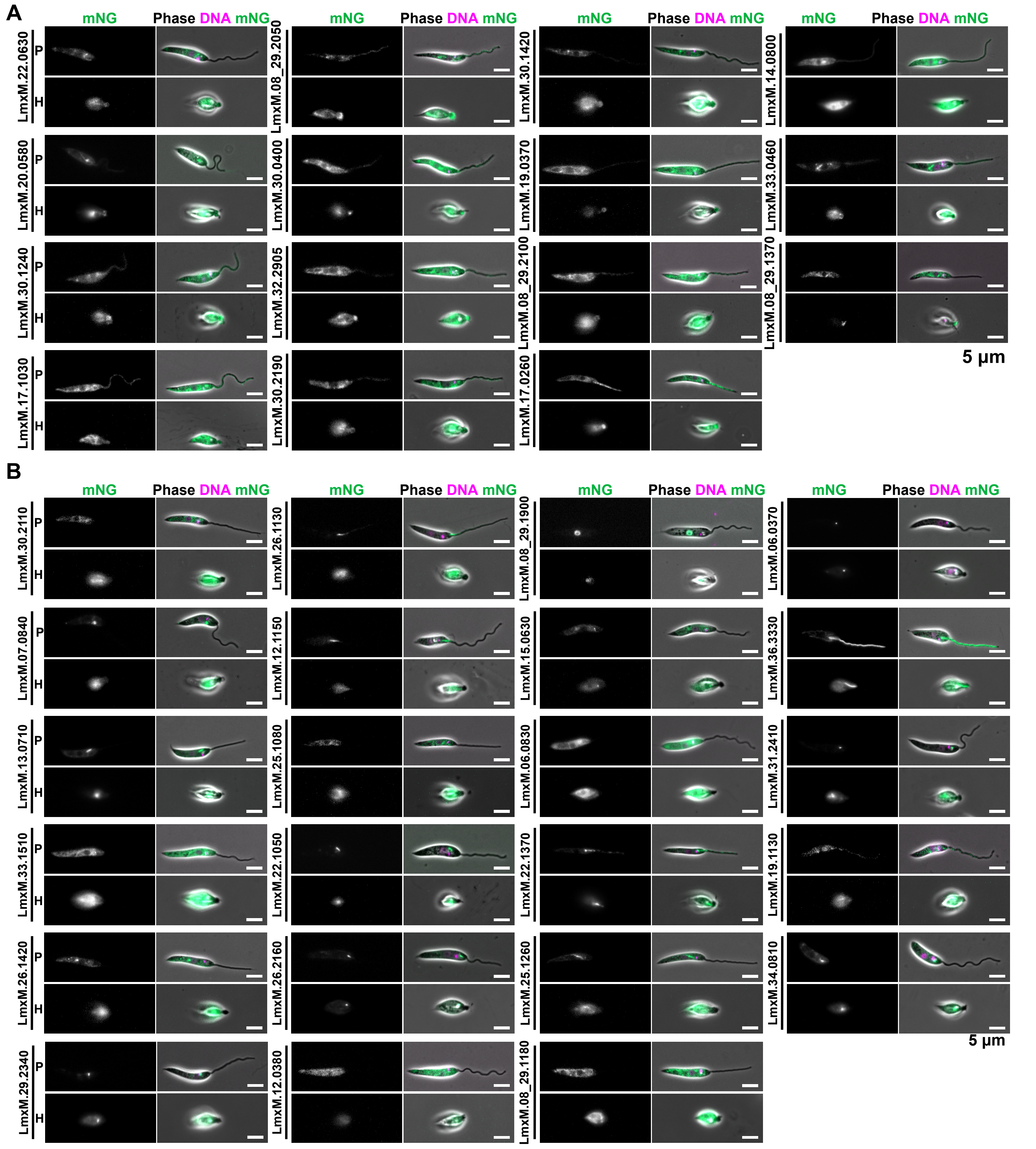

Figure S2. Tagging screen of the high confidence proteins identified through TurboID::KIAP3 proteomics, Related to Figure 1, Dataset S1, S2. (A and B) Localisation of mNG-tagged high confidence proteins in *L. mexicana in vitro* promastigotes and in vitro haptomonad cells showing a weak (A) or no signal (B) in the adhered flagellum of haptomonad-like cells. The minimum and maximum fluorescence intensity values were adjusted in Fiji to show the optimal signal for each protein. Images are representative of 1 biological repeat. For promastigotes, 50 cellls were imaged for each cell line. For mature in vitro haptomonads, weak adhered flagellum signal was observed in, 100% (n=6) for LmxM.22.0630, 100% (n=10) for LmxM.20.0580, 100% (n=5) for LmxM.30.1240, ~67% (n=6) for LmxM.17.1030, 100% (n=10) for LmxM.08_29.2050, 100% (n=7) for LmxM.30.0400, 100% (n=36) for LmxM.32.2905, 100% (n=5) for LmxM.30.2190, 100% (n=10) for LmxM.30.1420, 100% (n=13) for LmxM.19.0370, 100% (n=10) for LmxM.08_29.2100, 100% (n=10) for LmxM.17.0260, 100% (n=6) for LmxM.14.0800, 100% (n=5) for LmxM.33.0460, ~73% (n=11) for LmxM.08_29.1370. For mature in vitro haptomonads, no adhered flagellum signal was observed in, 100% (n=25) for LmxM.30.2110, 100% (n=5) for LmxM.07.0840, 100% (n=9) for LmxM.13.0710, 100% (n=5) for LmxM.33.1510, 100% (n=14) for LmxM.26.1420, 100% (n=4) for LmxM.29.2340, 100% (n=12) for LmxM.26.1130, 100% (n=6) for LmxM.12.1150, 100% (n=10) for LmxM.25.1080, 100% (n=9) for LmxM.22.1050, 100% (n=6) for LmxM.26.2160, 100% (n=6) for LmxM. 12.0380, 100% (n=5) for LmxM.08_29.1900, 100% (n=15) for LmxM.15.0630, 100% (n=6) for LmxM.06.0830, 100% (n=11) for LmxM. 22.1370, 100% (n=10) for LmxM.25.1260, 100% (n=9) for LmxM.08_29.1180, 100% (n=12) for LmxM.06.0370, 100% (n=7) for LmxM. 36.3330, 100% (n=10) for LmxM.31.2410, 100% for (n=8) for LmxM.19.1130, 100% (n=5) for LmxM.34.0810. The nucleus and kinetoplast DNA were stained with Hoechst 33342, and are shown in magenta. See also Figure 1 and Dataset S1 and S2.

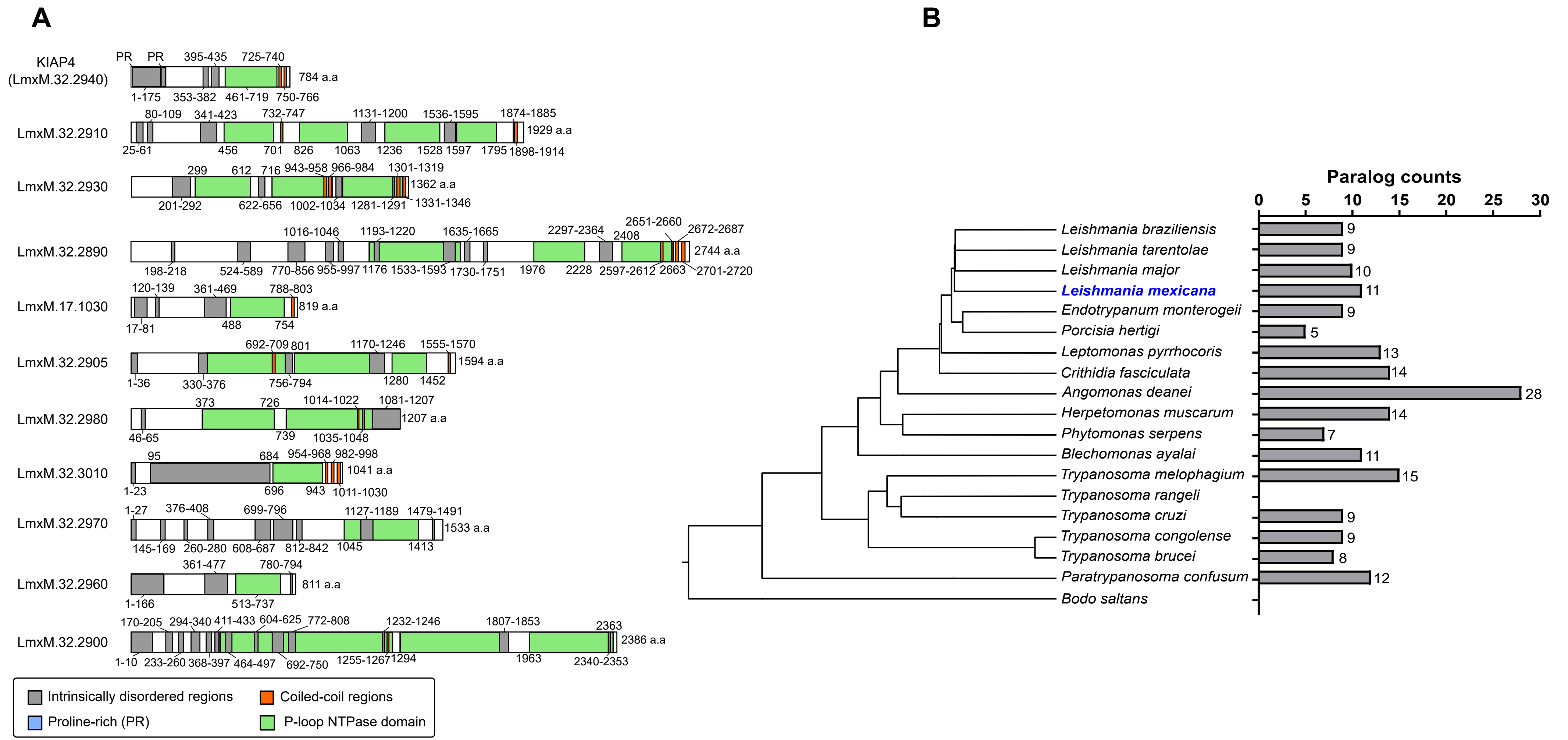

Figure S3. Adhesion Related NTPase-like Domain (ARND) gene family members have predicted coiled-coil regions and are conserved across many kinetoplastids, Related to Figures 1E, 2, 3, and Dataset S1, S2 and S3. (A) Domain architecture of KIAP4 and ten additional ARND gene family members in *Leishmania mexicana.* The ranges of the amino acid sequences for each domain are shown next to the coloured boxes, while the domain names are presented in the figure legend. The amino acid (a.a) sequence length for each protein is shown to the right of their structure. PR: Proline-rich residues of 10 amino acids, each. (B) A phylogenetic tree based on 18S rRNA showing the conservation of ARND gene family across kinetoplastids. Multiple sequence alignment was performed using MAFFT v7.511 web server with the L-INS-i iterative refinement method and default parameters. The maximum-likelihood species tree was inferred with PhyML (v3.0) using the TN93+G+I substitution model selected under the Bayesian Information Criterion with the built-in Smart Model Selection. There were 19 sequences and 2503 bases in the final dataset. The branch lengths do not represent evolutionary time. The numbers of ARND gene family paralogs in other kinetoplastids were identified through an exhaustive iterative BLAST search and are shown next to the bars. See also Figures 1E, 2, 3, and Dataset S1, S2 and S3.

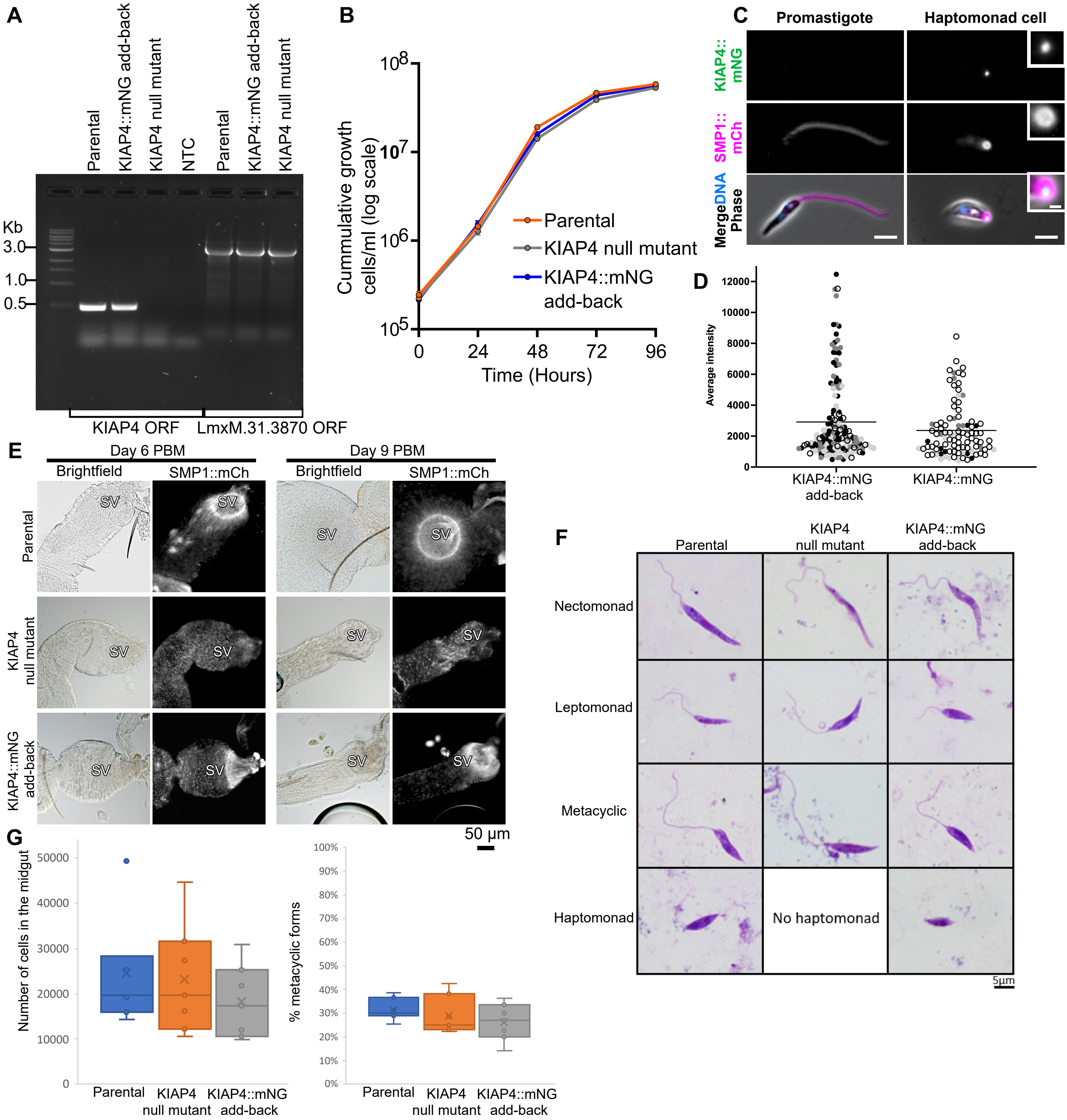

Figure S4. Confirmation of KIAP4 deletion and add-back and *in vitro* promastigote growth analysis, Related to Figure 4 and Movies S2-S4. (A) Confirmation of KIAP4 gene deletion and KIAP4::mNG add-back. Genomic DNA from the parental, KIAP4::mNG add-back, and KIAP4 null mutant cell lines expressing SMP1::mCh were analysed by PCR, alongside a negative control (NTC). DNA quality was assessed by amplifying ~3 Kb fragment of the *LmxM.31.3870* ORF from the same genomic DNA samples. NTC: nuclease-free water. (B) Growth curves of parental, KIAP4::mNG add-back, and KIAP4 null mutant cell lines from (A). The plot shows data from three independent experiments, while the error bars represent the mean ± SD. (C) Confirmation of KIAP4::mNG expression in the add-back cell line from by fluorescence microscopy. The nucleus and kinetoplast DNA were stained with Hoechst 33342 (blue). Scale bars: 5 μm, insets: 1 μm. (D) Quantification of the KIAP4::mNG fluorescent signal in the adhesion plaque of haptomonads generated from the endogenously tagged cell line (n=158) and the add-back (n=95). Shades of grey represent measurements from four different adhesion experiments, with the horizontal bar the mean of all measurements. No significant difference in signal intensity was seen, P = 0.07, unpaired t-test (E) Brightfield and fluorescence micrographs of dissected sand fly guts on day 6 (left panel) or 9 (right panel) PBM infected with parental, KIAP4 null mutant and KIAP4::mNG add-back cell lines from (A-C), expressing SMP1::mCh. SV: stomodeal valve. Representative images of infected midguts from two independent experiments are shown. (F) Nectomonad, leptomonad, and metacyclic forms were observed in the midguts on day 9 PBM in all cell lines. No haptomonds were found in KIAP4 deletion cell lines. Representativei mages from n = 7 infected midguts. (G) Quantification of the total number of parasites and percentage of metacyclics in the midgut on day 9 PBM. Boxes and whiskers indicate the median, upper and lower quartiles and 5th/95th percentiles. Crosses and dots indicate mean values and outliers, respectively. No significant differences in parasite number in the midgut and the percentage of metacyclics were observed (n = 7 infected midguts), P = 0.517 and 0.252, respectively, Kruskal-Wallis test. See also Figure 4 and Movies S2-S4.

Tables

Table S1. Primers used in this study.

**Leishmania mexicana tagging primers**	
**Primer name**	**Primer sequence (5' to 3')**
LmxM201190_UF	TGACATCCTTTTGAATCTCAACATCTTCCTgtataatgcagacctgctgc
LmxM201190_UR	CTCGTCTACCTTTCCGTTCACGGCCGTCATactacccgatcctgatccag
LmxM201190_5sg	gaaattaatacgactcactataggCACCCCTGTTTCTCTGTACAgttttagagctagaaatagc
LmxM313870_DF	GCTGCCGCTAAGATGCTGTTCAAGGTGAGCggttctggtagtggttccgg
LmxM313870_DR	CCCCGCACCGTCTCGCTCTCGCTTTCACCGccaatttgagagacctgtgc
LmxM313870_3sg	gaaattaatacgactcactataggGCGGGTGCGGTTGCCGCAGGgttttagagctagaaatagc
LmxM060370_DF	GAGAGGGATTGGATGCGCAATGCCTTCAAGggttctggtagtggttccgg
LmxM060370_DR	GTCAAAGAGTTCACAACTCGGGGACTGCGAccaatttgagagacctgtgc
LmxM060370_3sg	gaaattaatacgactcactataggGATACTGACGCAAGCTCACGgttttagagctagaaatagc
LmxM060830_DF	GAGGGTAGCGCTCCGGCACCGCCGCAGAAGggttctggtagtggttccgg
LmxM060830_DR	GGCGCCGGCAAACTCTCGGTCTTGAAGCCAccaatttgagagacctgtgc
LmxM060830_3sg	gaaattaatacgactcactataggGTGGCGGCGAAGGGGATGATgttttagagctagaaatagc
LmxM080930_DF	GTTGAGGCAGCGGAGCTGATCAAGGTGGGCggttctggtagtggttccgg
LmxM080930_DR	CGCTTCTTTCGGCCGTCTTCGCTTGTGTGCccaatttgagagacctgtgc
LmxM080930_3sg	gaaattaatacgactcactataggGCTTGTCCGGGTGCAGGGCAgttttagagctagaaatagc
LmxM08_291180_DF	TATGATAACATTTTGCGCCTAATGGAATCCggttctggtagtggttccgg
LmxM08_291180_DR	TCGTCGTCCATCACTCGGGCCACAACACCCccaatttgagagacctgtgc
LmxM08_291180_3sg	gaaattaatacgactcactataggAGGTTTTTTTTTGTGTGGGGgttttagagctagaaatagc
LmxM08_291370_DF	AATCTCATGAGCTACGACGACGCCTCTTACggttctggtagtggttccgg
LmxM08_291370_DR	ATGGGGGCTAGAAGCGAGGGGTGTGCACCGccaatttgagagacctgtgc
LmxM08_291370_3sg	gaaattaatacgactcactataggCAGCACATCCGCATGGCATAgttttagagctagaaatagc
LmxM08_291900_DF	GCCGACTCAAATGACCCATCTTCCCTCGCCggttctggtagtggttccgg
LmxM08_291900_DR	GAGCAGACATGCACACCGAACACCGTCAACccaatttgagagacctgtgc
LmxM08_291900_3sg	gaaattaatacgactcactataggCAAACGGACGGTCCGCAGCGgttttagagctagaaatagc
LmxM08_292050_DF	GAAATGTTCTCGCGGCAGAAAAGTTTCTCCggttctggtagtggttccgg
LmxM08_292050_DR	GCACCGCCTGTCGCGATGTGCGCCCACCCGccaatttgagagacctgtgc
LmxM08_292050_3sg	gaaattaatacgactcactataggAAAAACCACCTGTGTGCGCAgttttagagctagaaatagc
LmxM08_292100_DF	GCGCCAAGCAACGCCACCATATACGGGCTGggttctggtagtggttccgg
LmxM08_292100_DR	CCCGCGCATGTGTTTACAGGCACGTGAGCAccaatttgagagacctgtgc
LmxM08_292100_3sg	gaaattaatacgactcactataggCTAAACTGCTAGAGAAGCCGgttttagagctagaaatagc
LmxM120380_DF	CACCAACAGCAGCAGAAGCAGCGCCGCAGTggttctggtagtggttccgg
LmxM120380_DR	CAGAAGCCGGGAACGGTGGCCACACACACAccaatttgagagacctgtgc
LmxM120380_3sg	gaaattaatacgactcactataggCATGCAAAATGCTAAAAGAAgttttagagctagaaatagc
LmxM121150_DF	CTTCTAATGTCAGGCAGCAGCAGTGTAGCGggttctggtagtggttccgg
LmxM121150_DR	GGCGTTTGTGTCGGTCGGTGCAGGCGCGCCccaatttgagagacctgtgc
LmxM121150_3sg	gaaattaatacgactcactataggATGTGAGCTGGCGCTGGCTTgttttagagctagaaatagc
LmxM130710_DF	GAGGCGGTGAAGAACTTCTTGGATGATCTGggttctggtagtggttccgg
LmxM130710_DR	ACGAGAACCATCACTGTGCGCTGCGCTCCTccaatttgagagacctgtgc
LmxM130710_3sg	gaaattaatacgactcactataggCAAAACGGGAAAAAATGGGAgttttagagctagaaatagc

LmxM140800_DF	GTCTACAAGCCGCCGAGCAAGCGCGAGGCAggttctggtagtggttccgg
LmxM140800_DR	TCCCCGCGTTGATGGAGGCGCTTCGCTTCGccaatttgagagacctgtgc
LmxM140800_3sg	gaaattaatacgactcactataggTTCTCGAAAAAAATGCACGTgttttagagctagaaatagc
LmxM150440a_DF	GTCGAGGGCGCGCTGCGCGATGGTCAGGTAggttctggtagtggttccgg
LmxM150440a_DR	CGGCGGCCGTGCCACGAAGGGATGTCGCCGccaatttgagagacctgtgc
LmxM150440a_3sg	gaaattaatacgactcactataggGCGATATCTAGGGAATTCGTgttttagagctagaaatagc
LmxM150520_DF	AGGAGCGGCAACGGCAGCAAGGCAGACCTGggttctggtagtggttccgg
LmxM150520_DR	TCTCCTTGCGCCCCCACCGCCCTCCTCCCCccaatttgagagacctgtgc
LmxM150520_3sg	gaaattaatacgactcactataggCGGGAGGTAGTGGGAGACATgttttagagctagaaatagc
LmxM150630_DF	GCGCAGGGCAGCAGCAAGACCGCTGCAACGggttctggtagtggttccgg
LmxM150630_DR	CACTCATGCACACACGGCGCGTCTGTCTTTccaatttgagagacctgtgc
LmxM150630_3sg	gaaattaatacgactcactataggTGCTACGTCATTCTCTGCTGgttttagagctagaaatagc
LmxM161330_DF	GGCCTTTCACACAAAACGGACCAACGCGAGggttctggtagtggttccgg
LmxM161330_DR	TGCAGCAGTCCCTGGGGTATATGGAGTAGCccaatttgagagacctgtgc
LmxM161330_3sg	gaaattaatacgactcactataggAGACGCGCGCACAAGGACCAgttttagagctagaaatagc
LmxM170260_DF	CAGCAGCAGCAGCAAAGAACGAATCCGTCTggttctggtagtggttccgg
LmxM170260_DR	GCATCGTCACCACCGCCTCGACGAACCCATccaatttgagagacctgtgc
LmxM170260_3sg	gaaattaatacgactcactataggTAAGCCGCACAAATTGTGGCgttttagagctagaaatagc
LmxM171030_DF	CCGGCGGATTGCGTGCCCAAGACGTACATCggttctggtagtggttccgg
LmxM171030_DR	GAGGCGCAGCTTTCACCTCGCGTGCGACCCccaatttgagagacctgtgc
LmxM171030_3sg	gaaattaatacgactcactataggAGTGGGGTGTGTACCGGAGAgttttagagctagaaatagc
LmxM180070_DF	AACCGCCCAAGGCCACCTCCACGCCGTCGTggttctggtagtggttccgg
LmxM180070_DR	AAGAGCACAAAGAAGAGAGCCGCACAGAGGccaatttgagagacctgtgc
LmxM180070_3sg	gaaattaatacgactcactataggTGTGCCCGTTCAAATGCCTGgttttagagctagaaatagc
LmxM190370_DF	ATTCTCGCGTCGGCGCGTCTCTCCGACTCGggttctggtagtggttccgg
LmxM190370_DR	CGCACAAAGAGATGTGGAGAGGAGACGCCCccaatttgagagacctgtgc
LmxM190370_3sg	gaaattaatacgactcactataggATCGCGAGTGCTTCTTTTGCgttttagagctagaaatagc
LmxM191130_DF	GCACCAGCGGCGGCGCGACAGAAGCGGCGAggttctggtagtggttccgg
LmxM191130_DR	CAAAGCGAGAGCAGCAAGGAGCGAATACCGccaatttgagagacctgtgc
LmxM191130_3sg	gaaattaatacgactcactataggTGTGCGTGTCTGGGCGGAGGgttttagagctagaaatagc
LmxM200580_DF	CAAGCGAATGACGAGGACGGTGAACAGGGCggttctggtagtggttccgg
LmxM200580_DR	CATAGAAGCAGCAGTGTAGGGGATGGTCCGccaatttgagagacctgtgc
LmxM200580_3sg	gaaattaatacgactcactataggCAGACGGAACCACGTCGTTTgttttagagctagaaatagc
LmxM201040_DF	AACAAGGTGCGCATGGAGCAGCCGGCGTCGggttctggtagtggttccgg
LmxM201040_DR	ATCGAGAAAAAAGCGAAGACACCTCTACCGccaatttgagagacctgtgc
LmxM201040_3sg	gaaattaatacgactcactataggGTGGAGGACAAGCGACGATGgttttagagctagaaatagc
LmxM201050_DF	ACCTCACTTGCGTACGAGCTACCAGGCATAggttctggtagtggttccgg
LmxM201050_DR	CTGGTCCAATGCGTGTGCAGCAGCGACCCTccaatttgagagacctgtgc
LmxM201050_3sg	gaaattaatacgactcactataggACGCAGGTGGGAAGCTGCTCgttttagagctagaaatagc
LmxM210853_DF	TCTGANCNTNTCATCCCACCAACTCCGGAGggttctggtagtggttccgg
LmxM210853_DR	TTTTGCTTTCCTGGTGCGGCCGTGAAGCCTccaatttgagagacctgtgc
LmxM210853_3sg	gaaattaatacgactcactataggACTGTCGCTCCGGTATGAGGgttttagagctagaaatagc
LmxM220630_DF	TGGCGCCGGCACGCACCTCCGCGGGAGCGGggttctggtagtggttccgg
LmxM220630_DR	ACGCTAGCGTCCTCTCGGCAAAATGCGCCGccaatttgagagacctgtgc

LmxM220630_3sg	gaaattaatacgactcactataggCTCCTCTTCGCGACACCAGTgttttagagctagaaatagc
LmxM221050_DF	CGCACACATGCAGGTGGGCACACGCACATGggttctggtagtggttccgg
LmxM221050_DR	CTACCCCCGTGTCGCTACGATGAGGGTGCAccaatttgagagacctgtgc
LmxM221050_3sg	gaaattaatacgactcactataggACAGGAGGAGAGCGAGCGAGgttttagagctagaaatagc
LmxM221370_DF	GCGCCGCTCTTCAGTCCCACTAGCACGCATggttctggtagtggttccgg
LmxM221370_DR	CGACACACACACACGCACGCACGCACGCCGccaatttgagagacctgtgc
LmxM221370_3sg	gaaattaatacgactcactataggAGAAGCGAGGCTGTGCAATCgttttagagctagaaatagc
LmxM251080_DF	CCGCCCATATCCATGTGCACATGTGTTTCTggttctggtagtggttccgg
LmxM251080_DR	TACCCCGACGCTACGCTTAAGACTTTAGGAccaatttgagagacctgtgc
LmxM251080_3sg	gaaattaatacgactcactataggGCAGAGATATTGAAAGAGGAgttttagagctagaaatagc
LmxM251260_DF	TGCCACTGCTGCGACAGCGTCCATCTCTCGggttctggtagtggttccgg
LmxM251260_DR	AGAATGAAAGTCGTGAGAGAGAGGGAGGGGccaatttgagagacctgtgc
LmxM251260_3sg	gaaattaatacgactcactataggGGCAAGAAGACGATCAGCGGgttttagagctagaaatagc
LmxM261130_DF	CGCACTCATCCTTGGCATGCCTCTGCTCAGggttctggtagtggttccgg
LmxM261130_DR	AAATAGGGTTTTGGGGGAGGAGGCGCGGAGccaatttgagagacctgtgc
LmxM261130_3sg	gaaattaatacgactcactataggCCTCGCGATCCACTGAGGCAgttttagagctagaaatagc
LmxM261420_DF	AAGGTAACGCAGCGGGAGGTGCTGAAGTTTggttctggtagtggttccgg
LmxM261420_DR	CGCACACACACCTACTAAAACAAAGCGAAAccaatttgagagacctgtgc
LmxM261420_3sg	gaaattaatacgactcactataggCAAAACTTCAAGCGAAGCAGgttttagagctagaaatagc
LmxM262160_DF	TCACAGGAGGAGGTGAGCTGGGCAGCCTCGggttctggtagtggttccgg
LmxM262160_DR	GCGGTCTCCTCGCCAACAGAGATACAACCGccaatttgagagacctgtgc
LmxM262160_3sg	gaaattaatacgactcactataggAGCAGATCACGAGAGCGCGTgttttagagctagaaatagc
LmxM292340_DF	CTGGCAAAGCTATTTTCCAAGAAGAGCATAggttctggtagtggttccgg
LmxM292340_DR	CACGTGTCACCGATGGCATCCCCGCCTCCAccaatttgagagacctgtgc
LmxM292340_3sg	gaaattaatacgactcactataggAAAAAGAAACAACTGCGCAGgttttagagctagaaatagc
LmxM300400_DF	CGCATCTCTGGCAACGAAACGAAGAGTATGggttctggtagtggttccgg
LmxM300400_DR	GCGACGATGCGCCTACAGTTCTGTGTCCCGccaatttgagagacctgtgc
LmxM300400_3sg	gaaattaatacgactcactataggTTCGTGTCTGTGTGTTCACCgttttagagctagaaatagc
LmxM301420_DF	CGATGGGACTGGCGAAGCCTGAATAAAAGGggttctggtagtggttccgg
LmxM301420_DR	TTCAGATGCTGCTCCGCTGCCCGTCGCGACccaatttgagagacctgtgc
LmxM301420_3sg	gaaattaatacgactcactataggTTGCCCGATTCCTTCCGTCAgttttagagctagaaatagc
LmxM302110_DF	TCCGAAAATGCGGATTGGCGAAGGCGTAACggttctggtagtggttccgg
LmxM302110_DR	TACTTGCCATCTGACAGGTTCCTGCGGCCAccaatttgagagacctgtgc
LmxM302110_3sg	gaaattaatacgactcactataggCGTGGGCGGCAGCGCAGCTGgttttagagctagaaatagc
LmxM302140_DF	ACCGTAAACATGTTCGACCGCGGCGATTTTggttctggtagtggttccgg
LmxM302140_DR	GCGCACATGCACGCGCCCCCTCCTCCTCCTccaatttgagagacctgtgc
LmxM302140_3sg	gaaattaatacgactcactataggGAATGTCTCATGAGAAGAGAgttttagagctagaaatagc
LmxM302190_DF	AGGGAAGACCATCGACTTTTCCAGGACGCCggttctggtagtggttccgg
LmxM302190_DR	AACGCGACGTCCCTAGCGGGATCGAAACCAccaatttgagagacctgtgc
LmxM302190_3sg	gaaattaatacgactcactataggGACGTCACTCAAATGAGCCGgttttagagctagaaatagc
LmxM312410_DF	CGGATGCGGCGCCGGCTGCACCTCCTATGGggttctggtagtggttccgg
LmxM312410_DR	CCCGCACCCCTGCCCCCCCCCAAGTGCCGccaatttgagagacctgtgc
LmxM312410_3sg	gaaattaatacgactcactataggAGGGGCACAAGCCAAGGCTTgttttagagctagaaatagc
LmxM322890_DF	CCGCTTGGCGGCCCACCCCACTTGCGGGCAggttctggtagtggttccgg

LmxM322890_DR	ATAACGTAGGGTCAAGGCACACACAGGCCGccaatttgagagacctgtgc
LmxM322890_3sg	gaaattaatacgactcactataggGTTCAGAAATGTGTGTATGCgttttagagctagaaatagc
LmxM322905_DF	TTCCCCGACGTGCGCGAGCGCCGTAAGCGCggttctggtagtggttccgg
LmxM322905_DR	TCAACATATACACGGCCAGACGCAAAGCCGccaatttgagagacctgtgc
LmxM322905_3sg	gaaattaatacgactcactataggGCACTTTCCTCGGTCAGTGCgttttagagctagaaatagc
LmxM322910_DF	CCAGAGGGTCGCATCCCCAACACGTACATGggttctggtagtggttccgg
LmxM322910_DR	GCTATGTACGAATGTTCCTTCCCTTTTCCGccaatttgagagacctgtgc
LmxM322910_3sg	gaaattaatacgactcactataggTGTCGGCGGGGAGGAGGTTTgttttagagctagaaatagc
LmxM322930_DF	GGTGGTAGCGCGCCGGCCGTCTACAAGCGTggttctggtagtggttccgg
LmxM322930_DR	GGGGCGACAAGAAAAAAAAAATGATGTGTGccaatttgagagacctgtgc
LmxM322930_3sg	gaaattaatacgactcactataggGATCACCGGATATGCGATCCgttttagagctagaaatagc
LmxM322940_DF	ACGCAGCCGAAGGGCTACTCCATGGGCCGCggttctggtagtggttccgg
LmxM322940_DR	GGGTGATGTATTCACGACGTCGCCGCGCCAccaatttgagagacctgtgc
LmxM322940_3sg	gaaattaatacgactcactataggGCTTCCAAGCGACGAGAAGCgttttagagctagaaatagc
LmxM322960_DF	CCGAAGGCGGCCGCCCCGAGGGCGTACGTGggttctggtagtggttccgg
LmxM322960_DR	GCATTCGCGCATCGAATGTTCTCATCTCCTccaatttgagagacctgtgc
LmxM322960_3sg	gaaattaatacgactcactataggAAGAAAAAAGCGCCACCAAAgttttagagctagaaatagc
LmxM322980_DF	CACCATCACAGATCCTCCAAGGACAAGAAGggttctggtagtggttccgg
LmxM322980_DR	GCTACCTACAAACAGAGAGAGAGGCTTCCAccaatttgagagacctgtgc
LmxM322980_3sg	gaaattaatacgactcactataggGTGGCGCAATGGTGTGTTCAgttttagagctagaaatagc
LmxM323010_DF	GAGTGGAACATCAACTGCGGCGAGGGGCTCggttctggtagtggttccgg
LmxM323010_DR	GTCGAAATGAACCGCAGCGGTAGCGAAGTGccaatttgagagacctgtgc
LmxM323010_3sg	gaaattaatacgactcactataggTGGCGCACCCTCACGTCTGCgttttagagctagaaatagc
LmxM330460_DF	ATGGAGGAGCTCCGGAGGCTAGGGTTACTGggttctggtagtggttccgg
LmxM330460_DR	CTTGAAGTTTGAATGCAGTGGCAGCATCCTccaatttgagagacctgtgc
LmxM330460_3sg	gaaattaatacgactcactataggGTCTGTGAAGAGCAATGGCAgttttagagctagaaatagc
LmxM331510_DF	GAAGCGGAACTCTCGGCACTGAAAAAGCACggttctggtagtggttccgg
LmxM331510_DR	GAAAAAAAAAAGATGTCAACGCTGACGTGAccaatttgagagacctgtgc
LmxM331510_3sg	gaaattaatacgactcactataggGCGGCTGAAAGGGAGATGATgttttagagctagaaatagc
LmxM340810_DF	CTGGAAGATGTGCTGAGGACGGCGGGCTCGggttctggtagtggttccgg
LmxM340810_DR	ATCAAAGGAAAGCAGATGGGCGCCCACGGAccaatttgagagacctgtgc
LmxM340810_3sg	gaaattaatacgactcactataggAACGACATCCGGCAGAGCAAgttttagagctagaaatagc
LmxM341860_DF	TCGCTTTCTGACTCTTGCTCCCTGTCTCGCggttctggtagtggttccgg
LmxM341860_DR	CTCAGCCGCTCGCGCGTACGCCCCGTGCGCccaatttgagagacctgtgc
LmxM341860_3sg	gaaattaatacgactcactataggGTTAAGAGAAAACAAATACCgttttagagctagaaatagc
LmxM363330_DF	AAAAATGCGGCGCGCGCGAATGGTTACATGggttctggtagtggttccgg
LmxM363330_DR	GTATACAGCAGGCGTAAAGGCTGTCTGCCCccaatttgagagacctgtgc
LmxM363330_3sg	gaaattaatacgactcactataggGAAAGGCGTGCATAGTCATGgttttagagctagaaatagc
LmxM070840_DF	CCTCGGCCCCGCGGTGGCGAAGGACAAGGGggttctggtagtggttccgg
LmxM070840_DR	CGACGACTCACTGGAGCCCGAGTTGCGCCGccaatttgagagacctgtgc
LmxM070840_3sg	gaaattaatacgactcactataggGCTCCATCAATCACGGAAAGgttttagagctagaaatagc
LmxM270820_DF	CGCCGCTCCTCAGTGGCTTCAAAGAAGAGCggttctggtagtggttccgg
LmxM270820_DR	CAACCAAACATGCGTTTTGCATCTGATCCGccaatttgagagacctgtgc
LmxM270820_3sg	gaaattaatacgactcactataggGTGATACTTTGCCCGCTAAGgttttagagctagaaatagc

LmxM301240_DF	GTCAGCGGCGTCACCGCCACCGCGACACACggttctggtagtggttccgg
LmxM301240_DR	ATAAAAAACGAGAACACGCACCAACCCCACccaatttgagagacctgtgc
LmxM301240_3sg	gaaattaatacgactcactataggTAGTGGGGCATGATGTCAGTgttttagagctagaaatagc
LmxM.302115_DF	TCTGCCAGGGACAAGGGCAAGAAGCGCCACggttctggtagtggttccgg
LmxM.302115_DR	AGTCGCTCTCGCGAATCGGACGCTTACCCAccaatttgagagacctgtgc
LmxM.302115_3sg	gaaattaatacgactcactataggAGAGCTCCTACCACACGTCAgttttagagctagaaatagc
**Leishmania mexicana deletion primers**	
LmxM322940_UF	ATTTTCTCGCTAACGCGTTCCCTACTCCCCgtataatgcagacctgctgc
LmxM322940_5sg	gaaattaatacgactcactataggACTGCGACAAGAGAGGCGAAgttttagagctagaaatagc
LmxM322940_DR	GGGTGATGTATTCACGACGTCGCCGCGCCAccaatttgagagacctgtgc
LmxM322940_3sg	gaaattaatacgactcactataggGCTTCCAAGCGACGAGAAGCgttttagagctagaaatagc
LmxM322940_KOconfirmation forward	AGATGGCTGGGTCTGCCTAC
LmxM322940_KOconfirmation reverse	GTGGCAGGTCCTTGGCATTC
LmxM322940_AB_CT_Forward	ATATATaagcttATGAGCGCCATTGTTCCCCCT
LmxM322940_AB_CT_Reverse	ATATATactagtGCGGCCCATGGAGTAGCCCTT
LmxM313870 forward	atatattctagaATGGCGGAGCGTGTGTCTGTG
LmxM313870 reverse	atatatggatccCTAGCTCACCTTGAACAGCATC
**Trypanosoma congolense tagging primers**	
TcIL3000_2_1450 NT tagging forward	GAAAAAAGAAAAGAGAGAGAGAGGGAGTCTTTAGAACGCAAGTGAAAGGTGAGACCAAGGGAAGTTATAAAAGGAACAACgtataatgcagacctgctgc
TcIL3000_2_1450 NT tagging reverse	CATCCATTGGGGGCGTCATACCTTACATGTGGAAATCCCATATTTCCGTTACTTGTAAAACTGTACCCTCGGAAACTCATactacccgatcctgatcc
TcIL3000_0_08690 NT tagging forward	ACAAAGGAATATTGCACAAGGTGATACTCTGTGATAGGTGAAGTTTCCTAAGGACCAAAGAGAATCAGCAATCGCCATTTgtataatgcagacctgctgc
TcIL3000_2_1450 NT tagging reverse	GATATTTTTTTCAAGATTGAGCTTTTGCGCTGGAGTATCGACCCAGGGGGCGCGCCAGGTAAAGCCTGCGTTGTAGACATactacccgatcctgatcc
TcIL3000_0_08720 NT tagging forward	TCTATAACTTGGGGCTAGGAATCGAGCGAAGAGAAGGAGGGTAGCGAACCAGTTGGTTAGCCTTTTGGGAGACGAACAACgtataatgcagacctgctgc
TcIL3000_0_08720 NT tagging reverse	CGTTGTGCACCGTACGACGTAGTTCTTGCTCCGGCCGCTGCGCTACGCTGTGGATTAGCACCACCGATTTGGCTCCTCATactacccgatcctgatcc

Movie S1. Time-lapse movie showing the accumulation of KIAP4::mNG signal in the expanded region of the flagellum during adhesion of in vitro haptomonad cells. The timestamp in hours: minutes: seconds is shown at the top right of the movie for a playback of ~8.4 h at 1008x speed.

Movie S2. Real-time observation of the dissected thoracic midgut of the sand fly infected with the *L. mexicana* parental cell line on day 9 post blood meal (PBM).

Movie S3. Real-time observation of the dissected thoracic midgut of the sand fly infected with the *L. mexicana* KIAP4 null mutant cell line on day 9 PBM.

Movie S4. Real-time observation of the dissected thoracic midgut of the sand fly infected with the *L. mexicana* KIAP4::mNG add-back cell line on day 9 PBM.

Dataset S1. List of all proteins detected in the samples of adhered TurboID::KIAP3 and the parental cell lines, Related to Figures 1-4, S2 and S3, and Dataset S2. Identifications required precursor Qvalue <0.01 and protein Qvalue (Experiment) <0.01. The resulting protein level data were further filtered to require a minimum of two peptides per accepted protein. Data from two independent runs with two or three technical replicates are shown. Unique proteins with at least 4-fold change enrichment in the TurboID::KIAP3 compared with the parental cells and that were abundant (≥ 15 peptides) selected for tagging are shown in blue. KIAP2 (LmxM.30.0390) and KIAP3 (LmxM.20.1190) proteins are highlighted in purple. See also Dataset S2 and Figures 1-4, S2 and S3.

Dataset S2. List of proteins with at least 15 peptides and 4-fold change enrichment in the TurboID::KIAP3 (TIDK3) vs parental (C9T7) selected for tagging and localisation analysis, Related to Figures 1-4, S2 and S3, and Dataset S1 and Movies S1- S4. Members of the Adhesion Related NTPase-like Domain (ARND) gene family are shown in blue. KIAP3 (bait) is highlighted in purple. See also Figures 1-4, S2, S3 and Dataset S1 and Movies S1-S4.

Dataset S3. Summary table of p-loop NTPase domain analysis. Related to Figures 1-3, S3, and Dataset S2 The AlphaFold3 predicted structure for each ARND gene family protein was used as a Foldseek query, and the best match from a common model organism for each p-loop NTPase domain was identified. The Walker A motif from this sequence was then mapped onto the predicted domain in the ARND gene family member. The table includes the AlphaFold designation for the best match, the species name, e-value and sequence identity for that match, and the alignment to the Walker A motif. Many of the ARND gene family proteins contain multiple predicted p-loop NTPase domains, and this approach has been used to look at each predicted domain. The Predicted Aligned Error (PAE) plot has been included for each AlphaFold3 structural model. See also Figures 1-3, S3, and Dataset S2.

SI References

[1] Beneke T, Madden R, Makin L, Valli J, Sunter J, Gluenz E. A CRISPR Cas9 high-throughput genome editing toolkit for kinetoplastids. Vol. 4, Royal Society Open Science. 2017. p. 1–16.

[2] Coustou V, Guegan F, Plazolles N, Baltz T. Complete in vitro life cycle of Trypanosoma congolense: Development of genetic tools. PLoS Negl Trop Dis. 2010;4(3).

[3] Dean S, Sunter J, Wheeler RJ, Hodkinson I, Gluenz E, Gull K. A toolkit enabling efficient, scalable and reproducible gene tagging in trypanosomatids. Open Biol. 2015;5(1).

[4] Sunter JD, Yanase R, Wang Z, Catta-Preta CMC, Moreira-Leite F, Myskova J, et al. Leishmania flagellum attachment zone is critical for flagellar pocket shape, development in the sand fly, and pathogenicity in the host. Proc Natl Acad Sci U S A. 2019;116(13):6351–60.

[5] Halliday C. The role of the flagellum attachment zone in Leishmania mexicana flagellar pocket architecture. PhD thesis, Oxford Brookes Univ. 2021;(October).

[6] Sharma R, Sharad S, Minhas G, Sharma DR, Bhatia K, Sharma NK. DNA, RNA isolation, primer designing, sequence submission, and phylogenetic analysis. Basic Biotech Bioprocess Bioentrepreneursh. 2023;197–206.

[7] Owino BO, Yanase R, Marron AO, Moreira-Leite F, Vaughan S, Sunter JD. Discovery of a novel flagellar filament system underpinning Leishmania adhesion to surfaces. Curr Biol. 2025;35:1–11. https://doi.org/10.1016/j.cub.2025.04.064

[8] Yanase R, Moreira-Leite F, Rea E, Wilburn L, Sadlova J, Vojtkova B, et al. Formation and three-dimensional architecture of Leishmania adhesion in the sand fly vector. Elife. 2023;2022.

[9] Yanase R, Pružinová K, Owino BO, Rea E, Moreira-leite F, Taniguchi A, et al. Discovery of essential kinetoplastid-insect adhesion proteins and their function in Leishmania-sand fly interactions. Nat Commun. 2024;1–31. http://dx.doi.org/10.1038/s41467-024-51291-z

[10] Dean S, Sunter J. Light Microscopy in Trypanosomes: Use of Fluorescent Proteins and Tags. 2020;2116:367–83. http://link.springer.com/10.1007/978-1-0716-0294-2

[11] Cheah JS, Yamada S. A Simple Elution Strategy for Biotinylated Proteins Bound to Streptavidin Conjugated Beads using Excess Biotin and Heat. Biochem Biophys Res Commun. 2017;493(4):1522–7.

[12] Geoghegan V, Carnielli JBT, Jones NG, Saldivia M, Antoniou S, Hughes C, et al.

CLK1/CLK2-driven signalling at the Leishmania kinetochore is captured by spatially referenced proximity phosphoproteomics. Commun Biol. 2022;5(1):1–17.

[13] Demichev V, Szyrwiel L, Yu F, Teo GC, Rosenberger G, Niewienda A, et al. dia-PASEF data analysis using FragPipe and DIA-NN for deep proteomics of low sample amounts. Nat Commun. 2022;13(1).

[14] Hsiao Y, Zhang H, Li GX, Deng Y, Yu F, Valipour Kahrood H, et al. Analysis and Visualization of Quantitative Proteomics Data Using FragPipe-Analyst. J Proteome Res. 2024;

[15] Ritchie ME, Phipson B, Wu D, Hu Y, Law CW, Shi W, et al. Limma powers differential expression analyses for RNA-sequencing and microarray studies. Nucleic Acids Res. 2015;43(7):e47.

[16] Schindelin J, Arganda-Carrera I, Frise E, Verena K, Mark L, Tobias P, et al. Fiji - an Open platform for biological image analysis. Nat Methods. 2012;9(7).

[17] Mazo G. QuickFigures: A toolkit and ImageJ PlugIn to quickly transform microscope images into scientific figures. PLoS One. 2021;16(11 November):1–11. http://dx.doi.org/10.1371/journal.pone.0240280

[18] Volf P, Volfova V. Establishment and maintenance of sand fly olonies. J Vector Ecol. 2011;36(SUPPL.1):1–9.

[19] Sádlová J, Price HP, Smith BA, Votỳpka J, Volf P, Smith DF. The stage-regulated HASPB and SHERP proteins are essential for differentiation of the protozoan parasite Leishmania major in its sand fly vector, Phlebotomus papatasi. Cell Microbiol. 2010;12(12):1765–79.

[20] Walters LL. Leishmania Differentiation in Natural and Unnatural Sand Fly Hosts. J Eukaryot Microbiol. 1993;40(2):196–206.

[21] Rogers ME, Chance ML, Bates PA. The role of promastigote secretory gel in the origin and transmission of the infective stage of the infective stage of Leishmania mexicana by the sandfly Lutzomyia longipalpis. Parasitology. 2002;495–507.

[22] Shanmugasundram A, Starns D, Böhme U, Amos B, Wilkinson PA, Harb OS, et al. TriTrypDB: An integrated functional genomics resource for kinetoplastida. PLoS Negl Trop Dis. 2023;17(1):1–20.

[23] Altschul SF, Gish W, Miller W, Myers EW, Lipman DJ. Basic local alignment search tool. J Mol Biol. 1990;215(3):403–10.

[24] Zimmermann L, Stephens A, Nam SZ, Rau D, Kübler J, Lozajic M, et al. A Completely Reimplemented MPI Bioinformatics Toolkit with a New HHpred Server at its Core. J Mol Biol. 2018;430(15):2237–43. https://doi.org/10.1016/j.jmb.2017.12.007

[25] Ludwiczak J, Winski A, Szczepaniak K, Alva V, Dunin-Horkawicz S. DeepCoil - A fast and accurate prediction of coiled-coil domains in protein sequences. Bioinformatics.

2019;35(16):2790–5.
[26] Gabler F, Nam SZ, Till S, Mirdita M, Steinegger M, Söding J, et al. Protein Sequence Analysis Using the MPI Bioinformatics Toolkit. Curr Protoc Bioinforma. 2020;72(1):1–30.
[27] Hallgren J, Tsirigos KD, Damgaard Pedersen M, Juan J, Armenteros A, Marcatili P, et al. DeepTMHMM predicts alpha and beta transmembrane proteins using deep neural networks. bioRxiv. 2022;2022.04.08.487609. https://www.biorxiv.org/content/10.1101/2022.04.08.487609v1%0Ahttps://www.biorxiv.org/content/10.1101/2022.04.08.487609v1.abstract
[28] van Kempen M, Kim SS, Tumescheit C, Mirdita M, Lee J, Gilchrist CLM, et al. Fast and accurate protein structure search with Foldseek. Nat Biotechnol. 2024;42(2):243–6.
[29] Abramson J, Adler J, Dunger J, Evans R, Green T, Pritzel A, et al. Accurate structure prediction of biomolecular interactions with AlphaFold 3. Nature. 2024;630(8016):493–500.
[30] Wheeler RJ, Gluenz E, Gull K. The limits on trypanosomatid morphological diversity. PLoS One. 2013;8(11).
[31] Katoh K, Rozewicki J, Yamada KD. MAFFT online service: Multiple sequence alignment, interactive sequence choice and visualization. Brief Bioinform. 2019;20(4):1160–6.
[32] Kuraku S, Zmasek CM, Nishimura O, Katoh K. aLeaves facilitates on-demand exploration of metazoan gene family trees on MAFFT sequence alignment server with enhanced interactivity. Nucleic Acids Res. 2013;41(Web Server issue):22–8.
[33] Nguyen LT, Schmidt HA, Von Haeseler A, Minh BQ. IQ-TREE: A fast and effective stochastic algorithm for estimating maximum-likelihood phylogenies. Mol Biol Evol. 2015;32(1):268–74.
[34] Kalyaanamoorthy S, Minh BQ, Wong TKF, Von Haeseler A, Jermiin LS. ModelFinder: Fast model selection for accurate phylogenetic estimates. Nat Methods. 2017;14(6):587–9.
[35] Hoang DT, Chernomor O, von Haeseler A, Minh BQ, Vinh LS. UFBoot2: Improving the Ultrafast Bootstrap Approximation. Molecular biology and evolution. Mol Biol Evol. 2018;35(2):518–22.
[36] Lefort V, Longueville JE, Gascuel O. SMS: Smart Model Selection in PhyML. Mol Biol Evol. 2017;34(9):2422–4.
[37] Lefort, L., Longueville, J.-E., Olivier Gascuel O. Smart Model Selection in PhyML. Vol. 34. 2017. p. Mol. Biol. Evol. 2422–2424.
[38] Doleželová E, Kunzová M, Dejung M, Levin M, Panicucci B, Regnault C, et al. Cell-

based and multi-omics profiling reveals dynamic metabolic repurposing of mitochondria to drive developmental progression of Trypanosoma brucei. Vol. 18, PLoS Biology. 2020. 1–33 p.

[39] Denecke S, Malfara MF, Hodges KR, Holmes NA, Williams AR, Julia H, et al. Adhesion of Crithidia fasciculata promotes a rapid change in developmental fate driven by cAMP signaling. mSphere. 2024;9(10).

[40] Billington K, Halliday C, Madden R, Dyer P, Barker AR, Moreira-Leite FF, et al. Genome-wide subcellular protein map for the flagellate parasite Trypanosoma brucei. Nat Microbiol. 2023;8(3):533–47.
