# Supplementary material for: Identification of a conserved gene family with an essential role in Leishmania parasite–insect vector adhesion
Source: Proc Natl Acad Sci U S A. 2026 Jul 20;123(30):e2603653123. doi: 10.1073/pnas.2603653123 (PMC13416946; doi:10.1073/pnas.2603653123)
Supplement: Supplementary file 4 — Dataset S03 (PDF) [file pnas.2603653123.sd03.pdf]

Gene ID	Target	Description	Species	Identity (%)	E-value	Position in query	Walker A-motif (LmxM vs target)	Predicted Aligned Error (PAE)
LmxM.32.2940	AF-Q96L93-5-F1-model_v6	Kinesin-like protein KIF16B	Homo sapiens	13.8	7.43e-20	312-643	AGKG-RVE<br>+ G<br>GQTGSGKS	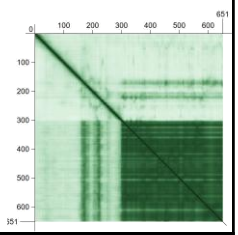
LmxM.32.2960	AF-A0A8M3AVN1-F1-model_v6	Kinesin-like protein KIF16B	Danio rerio	10.7	1.89e-18	318-643	SDATRRTQ<br><br>GQTGSGKS	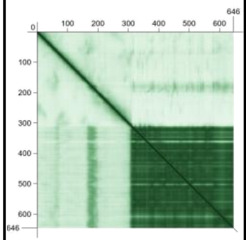
LmxM.17.1030	AF-A0A8M3AVN1-F1-model_v6	Kinesin-like protein KIF16B	Danio rerio	11.9	2.03e-17	472-810	DAGFSAAR<br>S+<br>GQTGSGKS	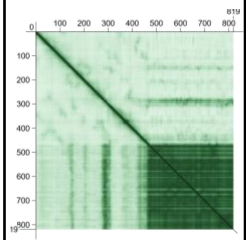
LmxM.32.3010	AF-A0A8M3AVN1-F1-model_v6	Kinesin-like protein KIF16B	Danio rerio	13.3	8.92e-18	683-1034	GRFDSPVW<br>G+ S<br>GQTGSGKS	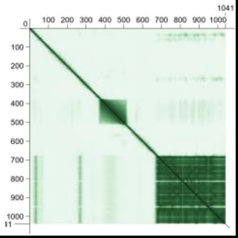
LmxM.32.2905	AF-A0A8M9PEJ5-F1-model_v6	Kinesin-like protein 6	Danio rerio	13.6	9.63e-16	1249-1577	EARGS---<br>GS<br>GQTGSGKS	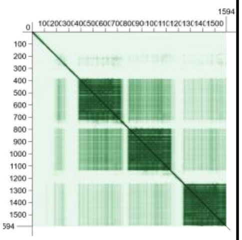
LmxM.32.2905	AF-P28738-F1-model_v6	Kinesin heavy chain isoform 5C	Mus musculus	15.1	3.28e-15	801-1177	EFT--EPT<br>T T<br>GQTSSGKT	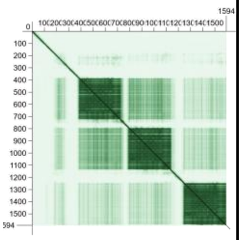
LmxM.32.2905	AF-Q9NS87-F1-model_v6	Kinesin-like protein KIF15	Homo sapiens	8.3	1.54e-14	362-759	DR------<br>+<br>GQTGSGKT	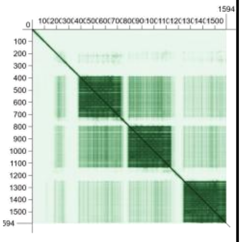

Gene ID	Target	Description	Species	Identity (%)	E-value	Position in query	Walker A-motif (LmxM vs target)	Predicted Aligned Error (PAE)
LmxM.32.2970	AF-B1AVY7-F1-model_v6	Kinesin-like protein KIF16B	Mus musculus	13.1	6.09e-5	1068-1492	NGNGRSTQ<br>G<br>GQTGSGKS	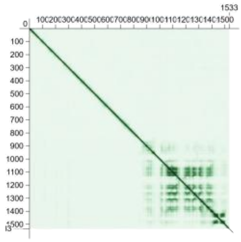
LmxM.32.2890	AF-A0A8M9PEJ5-F1-model_v6	Kinesin-like protein 6	Danio rerio	13.2	2.06e-17	1954-2284	SRP---TG<br>+<br>GQTGSGKS	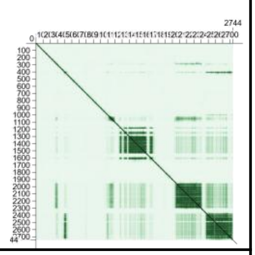
LmxM.32.2890	AF-O60333-F1-model_v6	Kinesin-like protein KIF1B	Homo sapiens	11.8	4.32e-17	1176-1616	STEKAHHA<br>A +<br>GQTGAGKS	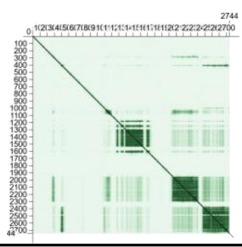
LmxM.32.2890	AF-Q8MQ20-F1-model_v6	Kinesin motor domain-containing protein	Caenorhabditis elegans	14.2	9.77e-14	2374-2724	DS-----S<br>S<br>GQTGSGKS	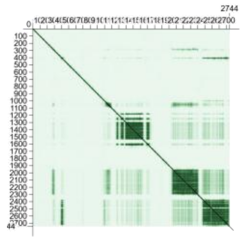
LmxM.32.2980	AF-Q965T6-F1-model_v6	Kinesin-like protein klp-20	Caenorhabditis elegans	13.2	9.77e-14	739-1084	VSPNEVNM<br><br>GQTGTGKT	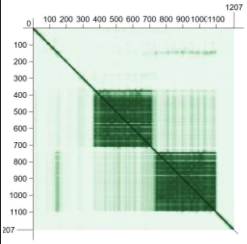
LmxM.32.2980	AF-Q12756-3-F1-model_v6	Kinesin-like protein KIF1A	Homo sapiens	14.2	7.28e-12	373-726	DGTTESRS<br>T +S<br>GQTGAGKS	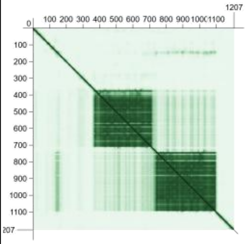
LmxM.32.2900	AF-P23678-2-F1-model_v6	Kinesin-like protein unc-104	Caenorhabditis elegans	12.9	2.60e-13	1963-2363	TESCDATS<br>++ + S<br>GQTGSGKS	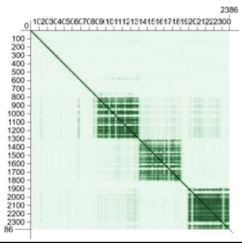

Gene ID	Target	Description	Species	Identity (%)	E-value	Position in query	Walker A-motif (LmxM vs target)	Predicted Aligned Error (PAE)
LmxM.32.2900	AF-Q94LW7-F1-model_v6	Kinesin-like protein KIN-4B	Arabidopsis thaliana	13.7	1.32e-12	1294-1843	ESAGENRA<br>G + GQTGSGKT	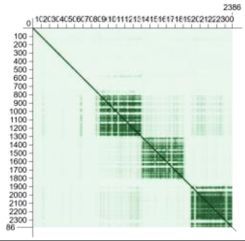
LmxM.32.2900	AF-Q15058-F1-model_v6	Kinesin-like protein KIF14	Homo sapiens	12.9	1.86e-14	434-1278	DT-25-CGRG-19-TA<br>G G<br>GQ---TGSG---KT	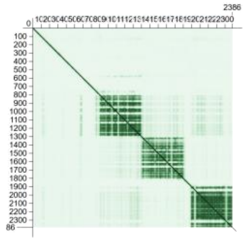
LmxM.32.2930	AF-G5EFQ4-F1-model_v6	Kinesin-like protein	Caenorhabditis elegans	14.1	2.83e-16	299-612	CG---SQP<br>+<br>GQTGSGKS	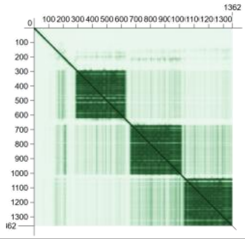
LmxM.32.2930	AF-G5ECF4-F1-model_v6	Kinesin-like protein	Caenorhabditis elegans	13.2	3.75e-15	1035-1349	--------<br><br>GQTGTGKT	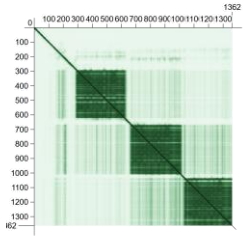
LmxM.32.2930	AF-P46873-2-F1-model_v6	Osmotic avoidance abnormal protein 3	Caenorhabditis elegans	13.1	1.53e-10	716-992	--------<br><br>GQTGSGKT	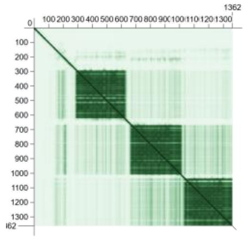
LmxM.32.2910	AF-A0A8M3AVN1-F1-model_v6	Kinesin-like protein KIF16B	Danio rerio	10.4	2.18e-15	431-756	DDGKAVGA<br>+ +<br>GQTGSGKS	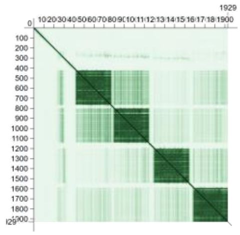
LmxM.32.2910	AF-A0A8M6YZ95-F1-model_v6	Kinesin-like protein	Danio rerio	13.4	8.74e-14	1594-1885	DVGGS---<br>GS<br>GHTGSGKT	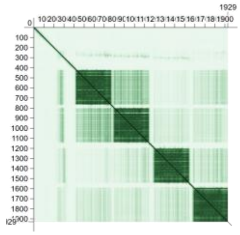

Gene ID	Target	Description	Species	Identity (%)	E-value	Position in query	Walker A-motif (LmxM vs target)	Predicted Aligned Error (PAE)
LmxM.32.2910	AF-AAF-Q965T6-F1-model_v6	Kinesin-like protein klp-20	Caenorhabditis elegans	14.2	3.46e-15	805-1128	DST-----A<br>T<br>GQTGTGKT	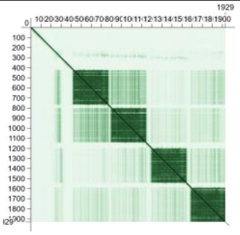
LmxM.32.2910	AF-P46873-2-F1-model_v6	Osmotic avoidance abnormal protein 3	Caenorhabditis elegans	12.7	1.04e-9	1236-1528	S--------<br><br>GQTGSGKT	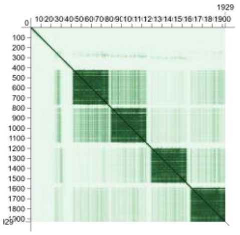

Dataset S3. Summary table of p-loop NTPase domain analysis. The AlphaFold3 predicted structure for each ARND gene family protein was used as a Foldseek query and the best match from a common model organism for each p-loop NTPase domain was identified. The Walker A motif from this sequenced was then mapped onto the predicted domain in the ARND gene family member. The table includes the AlphaFold designation for the best match, the species name, e-value and sequence identity for that match, and the alignment to the Walker A motif. Many of the ARND gene family proteins contain multiple predicted p-loop NTPase domains and this approach has been used to look at each predicted domain. The Predicted Aligned Error (PAE) plot has been included for each AlphaFold3 structural model.
